# High‐resolution single‐cell transcriptomic survey of cardiomyocytes from patients with hypertrophic cardiomyopathy

**DOI:** 10.1111/cpr.13557

**Published:** 2023-09-28

**Authors:** Jiansen Lu, Jie Ren, Jie Liu, Minjie Lu, Yueli Cui, Yuhan Liao, Yuan Zhou, Yun Gao, Fuchou Tang, Jizheng Wang, Shuiyun Wang, Lu Wen, Lei Song

**Affiliations:** ^1^ College of Life Sciences, Biomedical Pioneering Innovation Center Ministry of Education Key Laboratory of Cell Proliferation and Differentiation Beijing China; ^2^ Beijing Advanced Innovation Center for Genomics, College of Life Sciences Peking University Beijing China; ^3^ Peking‐Tsinghua Center for Life Sciences, Academy for Advanced Interdisciplinary Studies Peking University Beijing China; ^4^ State Key Laboratory of Cardiovascular Disease, Fuwai Hospital, National Center for Cardiovascular Diseases Chinese Academy of Medical Sciences and Peking Union Medical College Beijing China; ^5^ Department of Cardiovascular Surgery, Fuwai Hospital, National Center for Cardiovascular Diseases Chinese Academy of Medical Sciences and Peking Union Medical College Beijing China; ^6^ Cardiomyopathy ward, Fuwai Hospital, National Center for Cardiovascular Diseases, Chinese Academy of Medical Sciences and Peking Union Medical College Beijing China; ^7^ National Clinical Research Center for Cardiovascular Diseases, Fuwai Hospital, National Center for Cardiovascular Diseases Chinese Academy of Medical Sciences and Peking Union Medical College Beijing China

## Abstract

Hypertrophic cardiomyopathy (HCM) is a common inherited cardiovascular disease, which can cause heart failure and lead to death. In this study, we performed high‐resolution single‐cell RNA‐sequencing of 2115 individual cardiomyocytes obtained from HCM patients and normal controls. Signature up‐ and down‐regulated genes in HCM were identified by integrative analysis across 37 patients and 41 controls from our data and published human single‐cell and single‐nucleus RNA‐seq datasets, which were further classified into gene modules by single‐cell co‐expression analysis. Using our high‐resolution dataset, we also investigated the heterogeneity among individual cardiomyocytes and revealed five distinct clusters within HCM cardiomyocytes. Interestingly, we showed that some extracellular matrix (ECM) genes were up‐regulated in the HCM cardiomyocytes, suggesting that they play a role in cardiac remodelling. Taken together, our study comprehensively profiled the transcriptomic programs of HCM cardiomyocytes and provided insights into molecular mechanisms underlying the pathogenesis of HCM.

## Graphical abstract

In this study, we performed high‐resolution single‐cell RNA‐sequencing of individual cardiomyocytes obtained from HCM patients and normal controls. Combining with the published human single‐cell and single‐nucleus RNA‐seq datasets, we systematically investigated the key signature genes of HCM cardiomyocytes across 37 patients and 41 controls, which were further classified into gene modules by single‐cell co‐expression analysis. We also investigated the heterogeneity among individual cardiomyocytes and revealed five distinct clusters within HCM cardiomyocytes. Interestingly, we showed that some extracellular matrix (ECM) genes were up‐regulated in the HCM cardiomyocytes, suggesting that they play a role in ECM remodelling.

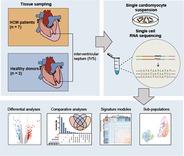

## 1 INTRODUCTION

Hypertrophic cardiomyopathy (HCM) is a common inherited cardiovascular disease characterized by left ventricular hypertrophy and is often caused by mutations in genes encoding sarcomere proteins.^1^, ^2^ Despite significant advances in understanding the genetic underpinnings of HCM, there remains an incomplete understanding of the molecular basis that underlies the cellular processes relevant to the pathogenesis of HCM. Single‐cell RNA‐sequencing (scRNA‐seq) have provided new opportunities to more deeply understand human diseases including cardiac diseases.^3^, ^4^ Recently, several scRNA‐seq studies on samples from both mouse cardiac hypertrophy models and HCM patients have been reported, however, most of these studies have focused on non‐cardiomyocyte cell types or analysed nuclei of cardiomyocytes rather than intact cardiomyocytes, as the large cell size of cardiomyocytes hinders the use of standard fluid‐ or droplet‐based devices.^5^, ^6^, ^7^, ^8^, ^9^ Since the cell mass of a cardiomyocyte is much larger than its nucleus, single‐cardiomyocyte RNA‐seq should be able to detect more transcripts per cell which should be complementary to these single‐nucleus RNA‐seq studies and may provide a more comprehensive characterization of the transcriptome of HCM cardiomyocytes.^10^ More recently, a single‐cardiomyocyte RNA‐seq study for human HCM samples has been reported, but the sequencing depth is still limited.^11^ Due to the heterogeneity of HCM, analysis based on limited cases may result in incomplete molecular characteristics. Therefore, integrative analyses are required to leverage the available resources and identify conserved transcription modules among different datasets. However, since these datasets were generated from different labs using various platforms, cross‐sample integration remains challenging.

To address these issues, in this study, we performed high‐resolution single‐cardiomyocyte RNA‐seq, investigated the transcriptional programs related to the remodelling of HCM cardiomyocytes, and integrated with published single‐cell and single‐nucleus RNA‐seq datasets of human HCM cardiomyocytes.

## 2 RESULTS

### 2.1 High‐resolution single‐cardiomyocyte RNA‐seq for HCM

**FIGURE 1:**
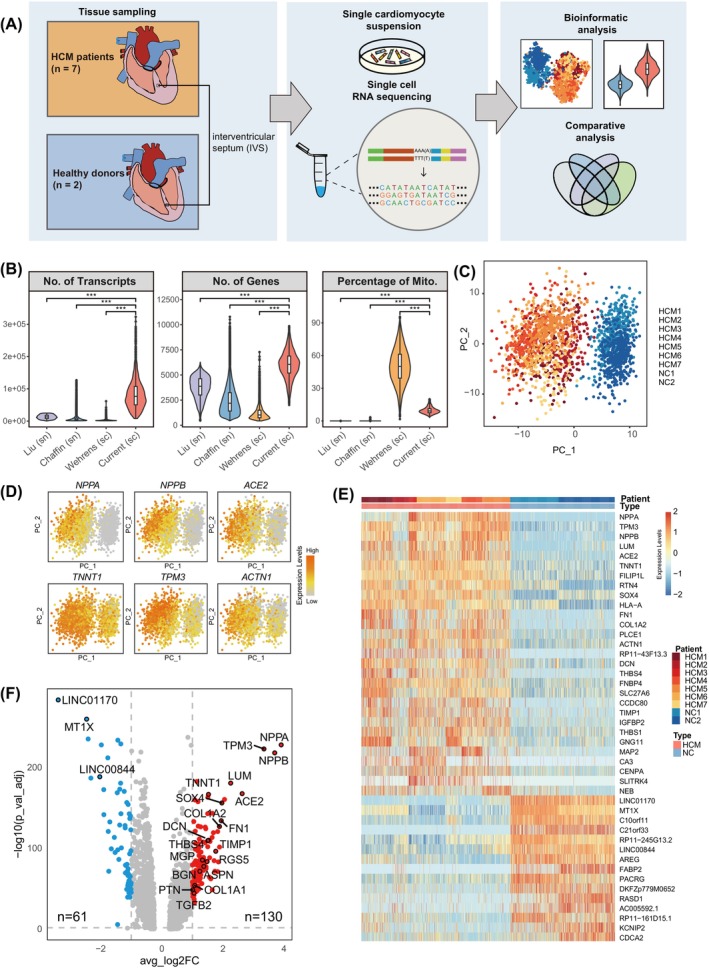
Single‐cardiomyocyte analysis reveals transcriptome alterations in HCM cardiomyocytes. (A) Schematic diagram of the summary of this study. (B) Comparison of quality control metrics between our data and published single‐cell or single‐nucleus RNA‐seq of cardiomyocytes from HCM. (C) The clustering result of cells from HCM patients and NC subjects was visualized with PCA. HCM, hypertrophic cardiomyopathy; NC, normal control. (D) The expression of representative cardiomyocyte markers (*TNNT1*, *TPM3* and *ACTN1*) and HCM markers (*NPPA*, *NPPB* and *ACE2*). (E) Heatmap showing significantly differentially expressed genes (DEGs) in CMs between HCM patients and NC subjects (adjusted *p*‐value < 0.05; fold change > 2). (F) Volcano plot of DEGs between HCM and NC related to (E).

To elucidate the transcriptional remodelling in cardiomyocytes of HCM, we performed high‐resolution scRNA‐seq on 2115 individual cardiomyocytes obtained from HCM patients (*n* = 7) and normal controls (NC, *n* = 2). After isolation of cardiomyocytes, scRNA‐seq was performed using our previously modified STRT‐seq method^12^ (Figure 1A). A total of 1971 high‐quality cells were retained for single‐cell analysis after stringent filtration based on the number of expressed genes and the fraction of mitochondrial counts (Figure S1a). We also eliminated potential cell contaminants to ensure the data quality (see also Methods, Figure S1b–g). As a result, an average of 6061 genes and 92,369 transcripts (counted as the unique molecular identifier, UMIs) were detected in each cell, which were significantly higher than other published single‐cardiomyocyte (one study) and single‐nucleus (two studies) RNA‐seq data^8^, ^9^, ^11^ (Figure 1B). Our data also exhibited a lower fraction of mitochondrial counts than the published single‐cardiomyocyte RNA‐seq data, which also indicated the high quality of our data (Figure 1B). By performing principal component analysis (PCA), cells from the HCM patients and the control subjects were clearly separated into two clusters (Figure 1C). The expression of typical cardiomyocytes markers such as *TNNT1* and *ACTN1*, and HCM markers such as *NPPA* and *NPPB* validated the cell type and the states (Figure 1D).

Examination of differentially expressed genes (DEGs) between the HCM and NC cardiomyocytes showed that 130 genes were significantly up‐regulated and 61 genes were down‐regulated in the cardiomyocytes of the HCM patients (adjusted *p*‐value < 0.05, fold change > 2, Figure 1E,F, Table S2). As expected, *NPPA* and *NPPB*, which are well‐known marker genes of HCM, were prominently up‐regulated (Figure 1D–F). The up‐regulation genes also included *TPM3*, *ACTN1* and *TNNT1*, which are related to cardiac muscle function and preferentially expressed in the foetal heart, indicating a reprogramming of gene expression in HCM from adult backward to foetal pattern^13^ (Figure 1C–E). By calculating Pearson's correlation coefficient of gene expression levels, we found co‐regulation of these genes among single cells within each individual (Figure S2). The causal genes of HCM showed relatively slight (such as *MYH7*, *MYBPC3*, *MYL2*, *TPM1*) or even not significantly difference (such as *MYL3*, *TNNT2*, *TNNI3* and *ACTC1*) when comparing cardiomyocytes from all HCM patients and the controls, which highlighted the heterogeneity between HCM patients and complexity of HCM pathogenesis (Figure S3a,b).

### 2.2 Biological pathways and regulatory networks of HCM cardiomyocytes

**FIGURE 2:**
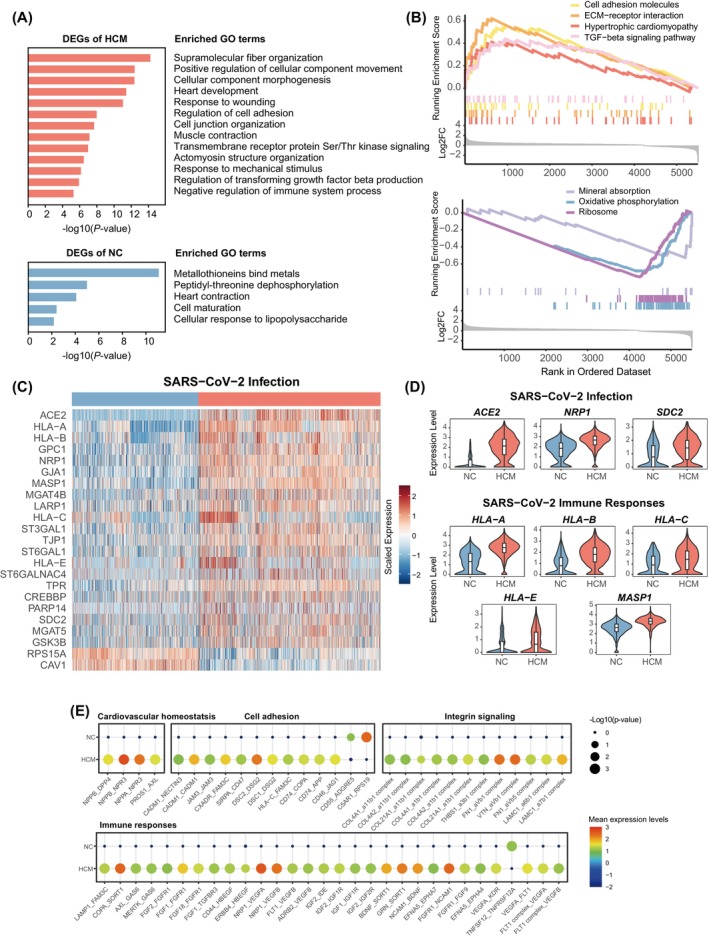
Enrichment analysis and pathway analysis in HCM. (A) Gene ontology (GO) enrichment result of DEGs between HCM and NC. (B) GSEA enrichment result of DEGs between HCM and NC in the KEGG signalling pathway. (C) Heatmap of differentially expressed genes between HCM and NC in the ‘SARS‐CoV‐2 Infection’ (R‐HSA‐9694516) pathway. (D) Representative genes from the ‘SARS‐CoV‐2 Infection’ and ‘SARS‐CoV‐2 activates/modulates innate and adaptive immune responses’ (R‐HSA‐9705671) pathway. (E) Ligand‐receptor pair expression levels among CMs from HCM patients and NC subjects.

To investigate which biological pathways were associated with the up‐regulated and down‐regulated genes in HCM cardiomyocytes, we performed pathway analysis including Gene Ontology (GO) enrichment and Gene Set Enrichment Analysis (GSEA). For the up‐regulated genes in HCM, the enriched GO terms included ‘heart development’, ‘muscle contraction’, ‘actin cytoskeleton organization’ and ‘regulation of cell adhesion’, which were also shown by the enriched GSEA pathway ‘hypertrophic cardiomyopathy’ and ‘cell adhesion molecules’ (Figure 2A,B). These enriched terms and pathways should reflect the up‐regulation of heart contraction, reactivation of some foetal genes, and reorganization of the cellular cytoskeleton and adhesion of the adaptive and maladaptive hypertrophy^6^ (Figure 2A,B, Table S4). Consistent with recent reports, we found that *ACE2* (angiotensin‐converting enzyme 2 receptor), a gene that encodes the main SARS‐CoV‐2 binding receptor, was markedly up‐regulated in HCM^14^ (Figure 1C–E). Interestingly, our analysis showed that a total of 21 genes in the ‘SARS‐Cov‐2 Infection’ pathway (R‐HSA‐9694516) were up‐regulated in HCM (Figure 2C). For example, we found that *NRP1*, which is another host factor of SARS‐Cov‐2 that can enhance SARS‐Cov‐2 infection, was also up‐regulated in HCM.^15^ Another gene *SDC2* involving in the cellular entry of SARS‐CoV‐2 was also up‐regulated.^16^ The up‐regulated genes also included several genes relating to the immune response of SARS‐CoV‐2 infection (e.g., *HLA* genes and *MASP1*) (Figure 2D). For the down‐regulated genes in the HCM cardiomyocytes, the enriched GO terms included ‘heart contraction’ and ‘cell maturation’, reflecting a foetal‐like reprogramming in HCM (Figure 2A). The GSEA analysis showed that the ‘oxidative phosphorylation’ and ‘ribosome’ pathways were down‐regulated, which have been shown to occur in the late stage of hypertrophy^6^ (Figure 2B). Interestingly, several metallothioneins (e.g., *MT1X*, *MT1E*, *MT3* and *MT2A*) that can bind metals and scavenge free radicals such as superoxide and hydroxyl radicals,^17^ were significantly down‐regulated in the HCM cardiomyocytes (Figure S3c,d), which may lead to their susceptibility to oxidative stress. Consistently, it has been reported that zinc supplementation can induce cardiac metallothionein and prevent diabetic cardiomyopathy in mice.^18^

Next, we performed the ligand‐receptor pair analysis to examine cell–cell interactions among cardiomyocytes. Our results revealed that several ligand‐receptor pairs were activated in the HCM cardiomyocytes, pointing to the up‐regulation of genes relating to cell adhesion and integrin signalling, cardiac homeostasis and growth factors (e.g., *VEGF*, *IGF*, *FGF* and *HBEGF* with their respective receptors functioning in cell growth and proliferation) during HCM progression (Figure 2E, Table S3).

**FIGURE 3:**
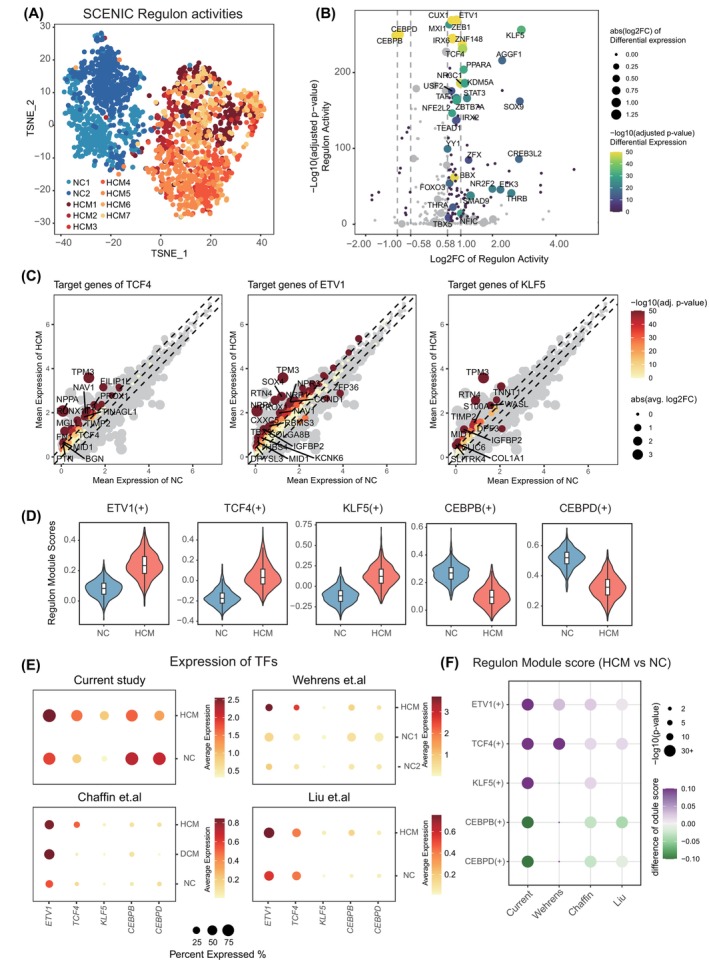
SCENIC analysis in HCM. (A) TSNE visualization with top 30 PCA components of SCENIC regulon AUC scores. (B) Differentially activated regulons between HCM and NC cardiomyocytes. (C) Expression of representative up‐regulated regulons in HCM cells, including *TCF4*, *ETV1* and *KLF5* regulons. (D) Gene module scores of target gene regulons of *TCF4*, *ETV1*, *KLF5*, *CEBPB* and *CEBPD* in NC and HCM, respectively. (E) Expression of *TCF4*, *ETV1*, *KLF5*, *CEBPB* and *CEBPD* in four HCM datasets, coloured by average expression levels. The size of the dot represented the percentage of expressed cells. (F) Relative gene module scores of target gene regulons of *TCF4*, *ETV1*, *KLF5*, *CEBPB* and *CEBPD* in four HCM datasets. Each dot was coloured by the relative module score between HCM and NC, the size of the dot represented the −log10(*p*‐value), two‐sided *t*‐test was used to determine the significance levels.

To explore the regulatory networks involved in HCM, single‐cell regulatory network inference, and clustering (SCENIC) analysis was performed to score the activity of gene regulatory networks of cardiomyocytes in HCM compared with NC.^19^, ^20^ We then used the regulon activity generated from SCENIC analysis to cluster all the cells and visualize them by t‐distributed stochastic neighbour embedding (TSNE), which showed that cardiomyocytes from HCM and controls were well separated (Figure 3A, Figure S4a), indicating that the gene regulatory network markedly altered in cardiomyocytes of HCM. Comparison of the regulon activity between HCM and NC identified activation of transcription factors (TFs) in HCM including *KLF5* which was associated with cardiomyopathy,^21^, ^22^ *ETV1* which is involved in atrial remodelling,^23^, ^24^ and *TCF4* which participates in the WNT signalling pathway (Figure 3B,C). TFs of *CEBPB* and *CEBPD*, which regulate genes involved in immune and inflammatory responses, were down‐regulated in HCM (Figure 3B, Figure S4b). A consistent distribution pattern for AUC scores of these regulon activities and the expression level of these TFs was found in HCM compared with control cardiomyocytes, which further validated the alteration of these TFs (Figure S4c). By analysing the target genes of these activated TFs, we found that the up‐regulated genes of HCM which regulating muscle contraction or relaxation, like *NPPA*, *TPM3, TNNT1* were included. Meanwhile, the regulon target gene module score (see Methods) of *ETV1*, *TCF4* and *KLF5* were up‐regulated in HCM, and that of *CEBPB* and *CEBPD* were down‐regulated (Figure 3D). Based on the SCENIC results, 105 differential activated regulons (DARs) were identified in HCM, among these activated regulons, 31 were overlapped with the 150 differential expressional transcriptional factors. Interestingly, only 14 DARs were identified in the controls with 2 DARs being overlapped with the 13 differential expressional transcriptional factors, again suggesting that HCM was characterized by dominant gene up‐regulation (Figure S4d). To validate the result of our SCENIC analysis, we calculated the expression of these TFs and the module score of related regulons in other published human single‐cell and single‐nucleus RNA‐seq data of cardiomyocytes from HCM,^8^, ^9^, ^11^ which revealed consistent patterns with our results (Figure 3E,F).

Together, our data revealed alterations of HCM cardiomyocytes in gene expression, biological pathways, ligand‐receptor pairs and TF networks.

### 2.3 Signature marker genes of HCM cardiomyocytes revealed by comparative analysis

**FIGURE 4:**
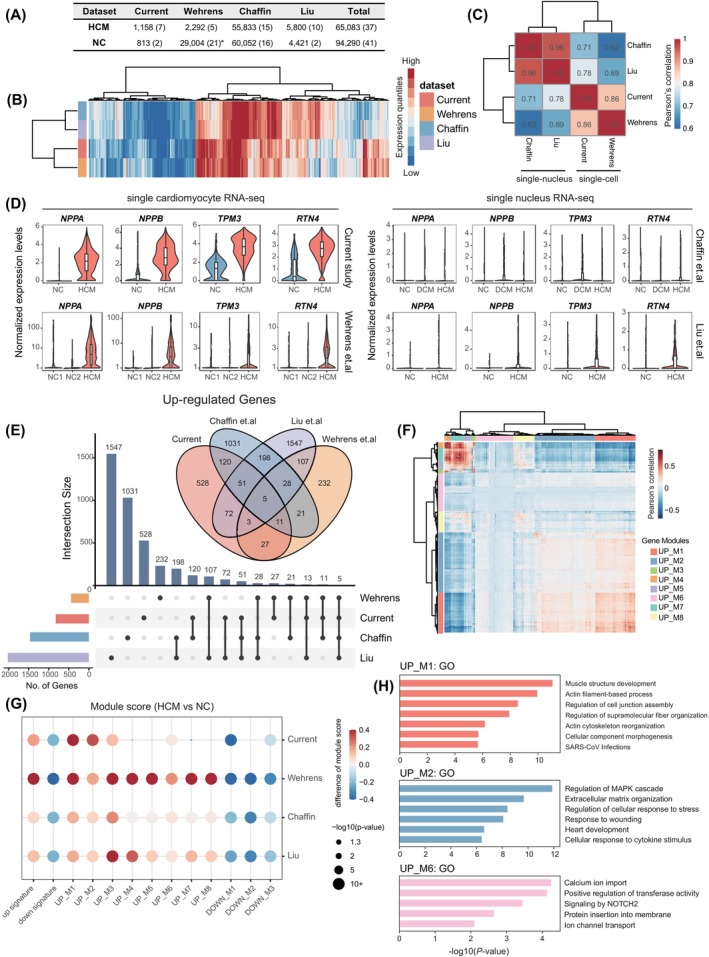
Comparative analysis revealed signature gene modules in HCM. (A) Summary of four datasets used in comparative analysis. The sequenced cell number of HCM and NC with the related number of samples in the parentheses were listed. *For the Wehrens et al. dataset, the NC data was derived from Wang et al. (1400 cells; *n* = 7) and Litvinukova et al. (27,604 cells; *n* = 14). (B) Heatmap showed the binned expression levels of the whole transcriptome of four datasets. (C) Pearson's correlation of binned expression levels of four datasets. (D) Normalized expression levels of *NPPA*, *NPPB*, *TPM3* and *RTN4* of four datasets, visualized with violin plots. (E) Venn diagram and upset plot of up‐regulated differential expressed genes identified from four datasets (adjuster *p*‐value < 0.05, fold change > 1.5), respectively. (F) Co‐expression heatmap of signature up‐regulated genes in cardiomyocytes from HCM of this study, visualized by Pearson's correlation coefficient between genes. Gene modules were classified by unsupervised hierarchical clustering. (G) Relative gene module scores of gene modules in four HCM datasets. Each dot was coloured by the relative module score between HCM and NC, the size of the dot represented the −log_10_(*p*‐value), two‐sided *t*‐test was used to determine the significance levels. (H) Representative gene ontology enrichment results of signature up‐regulated gene module UP_M1, UP_M2 and UP_M6.

Given the strong heterogeneity and complex genetic background of HCM patients, analysis of materials from limited individuals from different ethics may result in incomplete and biased estimation of molecular characteristics of HCM. To this end, we tried to integrate all published single‐cell RNA‐seq datasets for HCM. As a result, a total of 4 datasets, including 2 single‐cardiomyocyte RNA‐seq (including this study) and 2 single‐nucleus RNA‐seq data were collected, which constituted a meta dataset of 65,083 cells (nuclei) from 37 HCM patients and 94,240 cells (nuclei) from 41 normal controls (Figure 4A).^8^, ^9^, ^11^ To investigate batch effects among datasets derived from various patients and platforms, consensus genes from different datasets (*n* = 16,392) were classified into 10 groups based on their expression levels and used to perform unsupervised hierarchical clustering (Figure 4B). We showed that the overall distributions of gene expression were very different between single‐cardiomyocyte and single‐nucleus RNA‐seq data, which was also consistent with the higher Pearson's correlation coefficients within the two groups (Figure 4C). And we demonstrated that this difference may result in preference when performing differentially expression analysis. For example, the prominent up‐regulation of known HCM marker genes (including *NPPA*, *NPPB*, *TPM3* and *RTN4*) in HCM compared with NC were only captured in the two single‐cardiomyocyte RNA‐seq datasets while showing slight differences in the two single‐nucleus RNA‐seq datasets (Figure 4D). In addition, we also found that the sensitivity of DEGs identification may be reduced in low depth scRNA‐seq data, as the DEGs identified by Wehrens et al.^11^ were generally expressed with higher levels than DEGs identified by other datasets (Figure S5). Together, these results demonstrated the difference between scRNA‐seq and snRNA‐seq when applied to cardiomyocytes, which also highlight the advantages of high‐resolution scRNA‐seq data.

To overcome the batch effects among datasets, we then chose to perform multiple comparisons between DEGs identified by each dataset (adjusted *p*‐value < 0.05; fold change > 1.5), aiming to find common features of HCM cardiomyocytes. Only DEGs nominated by at least two datasets were remained for further analysis. In this way, we identified 653 up‐regulated and 185 down‐regulated genes as the signature marker genes of HCM. (Figure 4E; Figure S6c; Table S5). To reveal the modularity of these marker genes, we performed co‐expression analysis across single cells and identify gene modules for each dataset, respectively (Figure 4F; Figure S6a,d; Table S6). Benefiting from our high‐resolution scRNA‐seq data, 8 robust modules were identified among the up‐regulated signature genes, which was more than in other datasets. By calculating the differential module scores in these 8 modules in HCM compared with NC for each dataset, we observed that UP_M1 and UP_M2 were the most pervasive altered modules in all datasets, which mainly enriched in pathways such as ‘muscle structure development’ and ‘regulation of MAPK cascade’. And we also found that the UP_M6 was the most conserved module among datasets which was related to calcium ion import, indicating the dysregulation of ion channels in HCM (Figure 4G,H, Figure S6b). Three modules of down‐regulated signature genes were also identified in our data (Figures S6c–e and S7). Together, by integrating all available resources of scRNA‐seq data for human HCM, our results provided a comprehensive molecular feature profiling of cardiomyocytes from HCM.

### 2.4 Heterogeneity and subpopulation of cardiomyocytes from HCM

**FIGURE 5:**
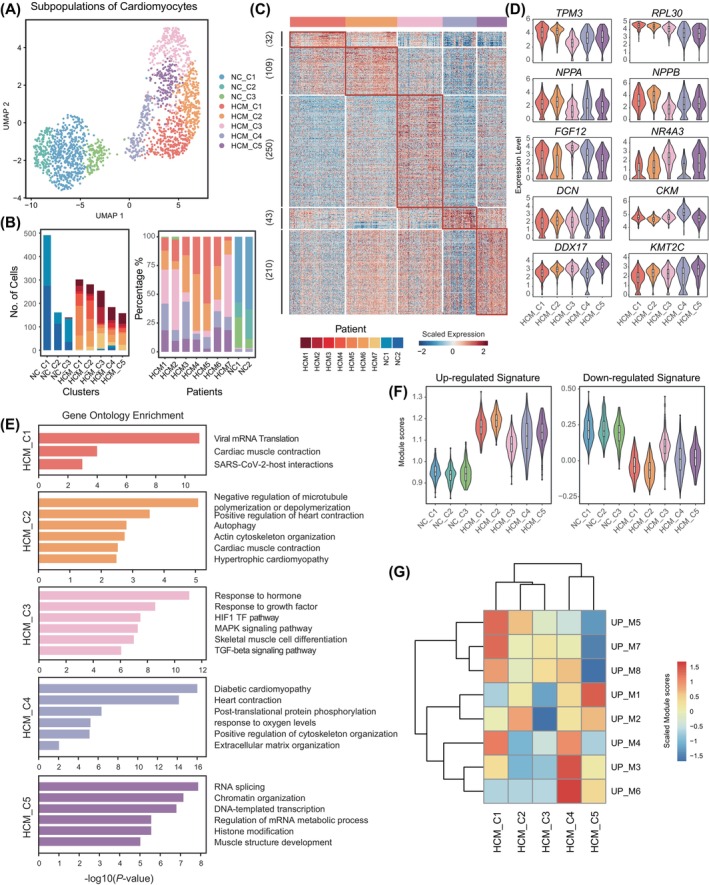
Heterogeneity revealed by subpopulations of cardiomyocytes from HCM. (A) UMAP1 showed the subpopulation of cardiomyocytes from HCM and NC, cells were coloured by clusters. (B) Summary of the individual information of each cell for each cluster. (C) Heatmap showed the differential expressed gene between the five clusters from HCM (adjusted *p*‐value < 0.05; log_2_(fold change) > 0.5). (D) The violin plot showed the normalized expression levels of representative DEGs between HCM subpopulations. (E) Representative gene ontology results of DEGs of HCM subpopulations related to (D). (F) Module score of signature up‐ and down‐regulated genes in all clusters of NC and HCM. (G) Heatmap showed the module score of signature up‐regulated modules in HCM subpopulations.

We next explored the cell heterogeneity of the cardiomyocytes. After the correction of batch effects derived from different individuals by the Harmony algorithm, five clusters of the HCM cardiomyocytes and three clusters of the NC cardiomyocytes were identified, respectively (Figure 5A,B). Differential expression analysis revealed significant gene expression differences among subpopulations (Figure 5C; Table S7). GO analysis for the differentially expressed genes of each HCM cluster showed that, cluster 1 was characterized by ribosome genes (e.g., *RPL30*, *RPL23A*) and enriched in the ‘Viral mRNA Translation’ pathway and ‘SARS‐CoV‐2‐host interactions’ (*RPS25*, *UBB*), which pointed to its association with the SARS‐CoV‐2 susceptibility of HCM patients. Cluster 2 exhibited the highest levels of canonical HCM markers *NPPA* and *NPPB* among all five clusters, and was relatively enriched in the ‘hypertrophic cardiomyopathy’ and ‘cardiac muscle contraction’ pathways. Cluster 3 showed activation of the growth factor pathway, including the high expression of *FGF12*. Cluster 4 was enriched in genes of the ‘heart contraction’ (e.g., *ACTC1*, *MYL3* and *ACTA1*) and ‘diabetic cardiomyopathy’ (e.g., *TNNI3*, *MYH7* and *GAS6*). It also enriched in genes related to energy homeostasis, such as *CKB*, *CKM* and mitochondrial genes, which were important markers for myocardial infarction,^25^ indicating a relationship between HCM and myocardial infarction. Additionally, Cluster 5 was related to RNA processing (e.g., *DDX17* and *RNPC3*) and chromatin organization (e.g., *KMT2C* and *KDM5A*), suggesting unknown mechanisms involved in HCM that should be further investigated (Figure 5D,E).

To further reveal the different usage of HCM clusters in transcriptional programs, we calculated the module score of previously identified signature gene modules for each cluster. We showed that cluster 2 exhibited the highest score of up‐regulated signature genes, consistent with its high‐level expression of HCM markers (Figure 5F). The preferential activation of gene modules in HCM clusters was consistent with their characteristics. For example, C1 showed the highest score of the ribosome module (UP_M7), and the modules related to muscle and heart development (UP_M1 and UP_M2) were both activated in C2 (Figure 5G). Taken together, these results showed the heterogeneity of cardiomyocytes from HCM.

### 2.5 Up‐regulation of ECM genes in cardiomyocytes of HCM

**FIGURE 6:**
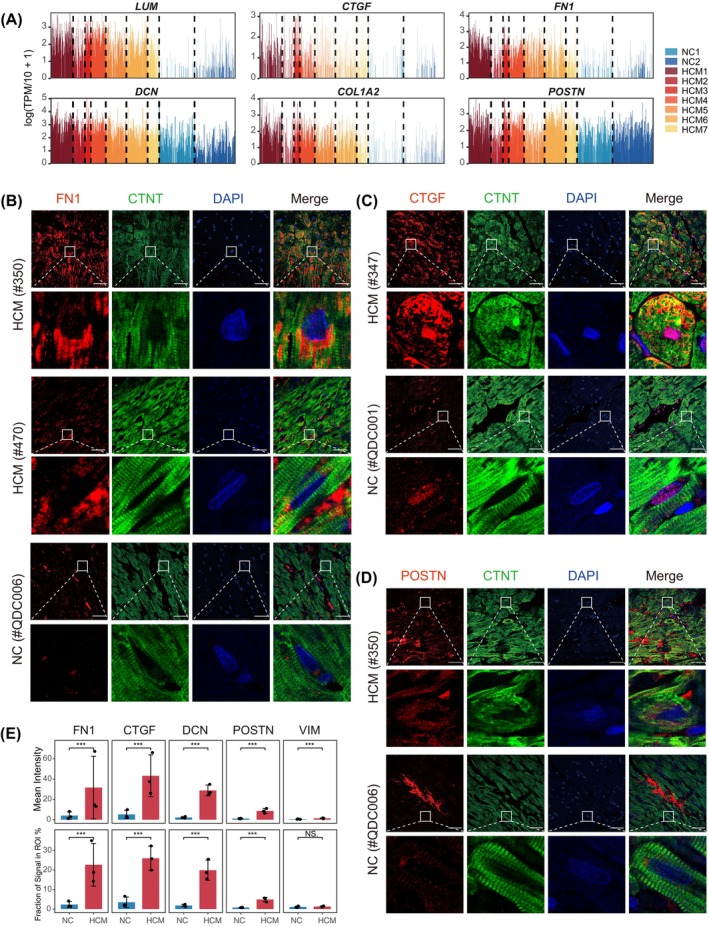
Up‐regulation of ECM genes in cardiomyocytes of HCM. (A) Expression of representative ECM genes in cardiomyocytes of HCM and NC in this study. (B–D) Immunofluorescence staining of FN1 (B), CTGF (C), POSTN (D) in HCM and NC. Individual cardiomyocytes were defined using CTNT (green) and DAPI (blue). FN1, CTGF and POSTN are ECM genes (red). Scale bar, 50 μm. (E) Quantitative statistic of immunofluorescence staining results. The mean fluorescence intensities and fractions of signals in the region of interest (ROI) were calculated. Each dot represented results from three representative fields from an individual.

Cardiac fibrosis, characterized by excessive accumulation of extracellular matrix components and deposition of collagen, is an important hallmark of HCM and contributes to impaired cardiac relaxation, cardiac sudden death and heart failure.^26^, ^27^, ^28^ Previous studies highlighted the central role of the activated myofibroblasts and TGF‐β signalling pathway in cardiac fibrosis.^26^ Unexpectedly, we found that many ECM genes, including *LUM*, *DCN*, *FN1*, *CTGF* and *COLIA2*, as well as the ‘extracellular matrix organization’ pathway, were prominently up‐regulated in all the subpopulations of HCM cardiomyocytes, especially in cluster 4 (Figure 6A–E and Figures 1E,F and 2A,B). We confirmed that a large proportion of up‐regulated DEG genes intersecting between our data and previous scRNA studies on both human DCM patients and mouse cardiac hypertrophy models were ECM genes and the ECM pathway was commonly activated in the HCM cardiomyocytes of published HCM scRNA‐seq or snRNA‐seq datasets (Figures S8 and S9).^5^, ^6^, ^7^, ^8^, ^9^, ^11^

To validate the up‐regulation of the ECM genes in HCM cardiomyocytes, we performed immunofluorescence staining of FN1, CTGF, DCN, POSTN and VIM on heart tissues from HCM patients and NC (*n* = 3 for each group) (Figure 6B–D, Figures S10 and S11). In the normal heart tissue, positive expression of all five genes can be seen in the interstitial region, while the cardiomyocytes largely displayed no or weak staining. In the HCM patients, the expression of POSTN was strongly increased in some interstitial regions, which was consistent with previous studies (Figure 6D, Figure S11); the expression of VIM also appeared to be slightly increased (Figures S10 and S11). The expressions of FN1, CTGF and DCN were evidently increased in the HCM heart tissues. Interestingly, in two HCM patients (Patient #347 and #350), we found that the elevated expressions of FN1 and CTGF were mainly located in the cytoplasm of the HCM cardiomyocytes, and the elevated expression of DCN was located both in the interstitial region and the cytoplasm (Figure 6B,C, Figure S10). Strong expression of FN1 was found in the perinuclear space of the hypertrophic cardiomyocytes, suggesting that it was accumulated in the endoplasmic reticulum (Figure 6B, Figure S11). CTGF displayed an intriguingly asymmetric pattern in the cytoplasm, and the expression was also found in the nuclei (Figure 6C, Figure S11). Another HCM patient (Patient #470) did not show such evident cytoplasm FN1 and CTGF gene expression patterns, and prominent FN1 expression was clearly located in the interstitial region (Figure 6B, Figure S11). To quantify the expression of these ECM genes within the cardiomyocytes, we limited the region of interest (ROI) for measurements based on the immunofluorescence signal of cardiac troponin T (CTNT), which selectively expressed inside the cardiomyocytes (Figure S11). As a result, the mean cardiomyocytic fluorescence intensities of FN1, CTGF and DCN were significantly elevated in HCM, with a fold change of 8.27, 8.38 and 12.86, respectively. The factions of signal in ROI of FN1, CTGF and DCN were also increased by 21.4%, 22.5% and 18.1%, respectively. Although POSTN was strongly elevated in the interstitial region in HCM patients, its expression in the cardiomyocytes was only slightly increased (fold change = 7.73; increased faction = 4.1%), which was consistent with the RNA expression data (fold change = 1.55, *p*‐value = 4.1e−13) (Figure 6E). We also performed immunofluorescence staining for FN1, CTGF and DCN in a double mutant knock‐in mouse HCM model (see Methods). However, the results showed that, despite that the mice clearly exhibited cardiac hypertrophy and fibrosis, the expressions of FN1 and CTGF were not elevated, while the DCN expression was slightly increased in the interstitial region (Figure S12, see Section 3).

Together, these results indicated that some ECM genes, that is, FN1 and CTGF, were up‐regulated and located in the cytoplasm of the cardiomyocytes in some HCM patients.

### 2.6 Validation in large bulk RNA‐seq dataset

Since cardiomyocytes constitute the majority masses of the myocardium, we supposed that the signature features of cardiomyocytes from HCM identified in this study could be applied to sequencing data from bulk samples, which may facilitate researches about the pathogenesis of HCM. To this end, we downloaded the large‐scale bulk RNA‐seq data from the Myocardial Applied Genomics Network (MAGNet, www.med.upenn.edu/magnet), which contains 28 HCM samples, 166 DCM samples, 6 PPCM (peripartum cardiomyopathy) samples and 166 non‐failing controls. PCA of these data revealed a clear separation of NC and other samples in the second principle component (PC2) (Figure S13a). We then sought to verify the expression of known HCM markers. As expected, both *NPPA* and *NPPB* were significantly elevated in all three groups compared with NC, while the *ACTN1* only significantly up‐regulated in HCM, and *ACTA1* was not increased in PPCM (Figure S13b). Analysis of signature gene sets revealed high correlations between pathological state and module score, as the signature up‐regulated genes were positively correlated with PC2 (Pearson's *r* = 0.64, *p*‐value = 2.2e−43), while signature down‐regulated genes were negatively correlated (Pearson's *r* = −0.85 *p*‐value = 2.3e−102) (Figure S13c,d). The score of each gene module also changed consistently, with UP_M1, UP_M2 and UP_M6 showing the most significant up‐regulation in HCM (Figure S13e). What's more, we also verified the expression of key TFs and their regulons identified by our SCENIC analysis in MAGNet dataset, which further confirmed our results (Figure S13f,g). These results suggested that the molecular mechanism identified in our single‐cardiomyocytes analysis could also be validated in larger experiments.

## 3 DISCUSSION

In this study, we used high‐resolution single‐cardiomyocyte RNA‐seq for comparing gene expression patterns of the HCM and NC cardiomyocytes. Combining with the published human single‐cell and single‐nucleus RNA‐seq datasets, we systematically investigated the key transcriptional alterations of HCM cardiomyocytes. We also addressed the heterogeneity of HCM cardiomyocytes by performing single‐cell clustering and analysing the preferential usage of gene modules among subpopulations. Our immunofluorescence staining results interestingly showed that some ECM genes, that is, FN1 and CTGF, were up‐regulated and located in the cytoplasm of the cardiomyocytes in some HCM patients.

With the rapid development of single‐cell RNA‐seq technologies, various approaches have been established and used in a wide range of fields. However, the differences across platforms, especially when applied to specialized cell types such as cardiomyocytes, were required to estimate. In this study, we demonstrated the distinct gene expression patterns between scRNA‐seq and snRNA‐seq data when comparing our data with other published human HCM datasets. Due to the large cell size of cardiomyocytes, the cytoplasm of cardiomyocytes may contain more mRNA molecules than other cell types, while these mRNAs may not be sufficiently captured by single‐nucleus methods. This was consistent with previous reports that snRNA‐seq may result in the underrepresentation of some cell types or depletion of a small set of genes.^29^, ^30^ However, snRNA‐seq of cardiomyocytes is easier to operate and can achieve higher throughput with fluid‐ or droplet‐based devices. Thus, these two methods are complementary.

Using high‐resolution scRNA‐seq, our results revealed the common gene expression changes of the HCM cardiomyocytes compared with other studies,^8^, ^11^ including the prominent up‐regulation of *NPPA*, *NPPB* and biological pathways such as ‘muscle contraction’ and ‘heart development’. Consistent with the previously reported elevated expression of *ACE2* in HCM^14^ and the potential link between HCM and COVID‐19,^31^ we showed that about 20 genes related to SARS‐Cov‐2 infection and immune response were up‐regulated in HCM. We also found that *ACE2* was co‐regulated with *NPPB* in our data. Using SCENIC analysis, several differential activated TFs between HCM and NC were identified, including three TFs (*ETV1*, *TCF4* and *KLF5*), which were validated in other datasets and large‐scale bulk RNA‐seq data. Interestingly, the target genes of *ETV1*, *TCF4* and *KLF5* also included *NPPA*, *TPM3*, *RTN4* and *TNNT1*, indicating a potential link between the TFs and marker genes of HCM. Further research about the underlying regulatory mechanism of these TFs and their target genes may provide more information about the pathogenesis of HCM.

Importantly, we further identify the conserved transcriptional programs across datasets by performing integrative analyses. To overcome the batch effects between datasets, a differential‐feature‐based strategy were used to identified common signatures of HCM cardiomyocytes. Moreover, our high‐resolution scRNA‐seq allowed us to further detect robust modules from these signature genes, which provided a more comprehensive understanding of the transcriptional programs in the HCM cardiomyocytes.

The heterogeneity of cardiomyocytes in both normal and disease status has been reported in recent single‐cell research. In this study, using our high‐resolution single‐cell RNA‐seq data, five distinct clusters of HCM cardiomyocytes were identified. Consistently, a cluster with high‐level expression of *NPPA* and *NPPB* (HCM_C2) was reported by two other studies^9^, ^11^ and the cluster showed response to growth factor (HCM_C3), featured by expression of *FGF12* was also identified in Liu et al.^9^ Additionally, clusters related to viral mRNA translation (HCM_C1), energy homeostasis (HCM_C4) and chromatin remodelling (HCM_C5) were also captured in our data, reflecting the advantages of high‐resolution data.

Previous bulk RNA‐seq studies on HCM have observed the up‐regulation of ECM genes in the HCM heart tissue; however, the cardiomyocyte and the myofibroblast were analysed in a mixed manner in these studies.^28^, ^32^, ^33^ Here, our scRNA‐seq data showed that the expression levels of some ECM genes were elevated in cardiomyocytes of HCM, which were confirmed by reanalyzing previous datasets. Our immunostaining results interestingly showed that the elevation of FN1 and CTGF were mainly located in the cytoplasm of the cardiomyocytes in two of three HCM patients. As the ECM genes generally do not re‐enter the cell after secretion, these results thus allowed us to conclude that these ECM genes were up‐regulated in the HCM cardiomyocytes, which validated the scRNA‐seq results. Consistent with our results, a previous study also reported the up‐regulation of CTGF in the cytoplasm of cardiomyocytes in dilated cardiomyopathy patients.^34^ Also, several studies have reported that the elevation of CTGF during mouse heart hypotrophy is mainly derived from cardiomyocytes.^34^, ^35^ However, ubiquitous or cardiomyocyte‐specific elimination of CTGF in mice does not yield a significant impact on the progression of cardiac fibrosis.^35^, ^36^, ^37^ The accumulation of FN1 in the cytoplasm of human HCM cardiomyocytes has not been previous reported. In two of three HCM patients, the expression of FN1 predominantly located in the cytoplasm of the cardiomyocytes, and it appeared to accumulate in the endoplasmic reticulum of HCM cardiomyocytes, which may increase endoplasmic reticulum stress in cardiomyocytes.^38^ In another HCM patient, in contrast, FN1 was clearly located in the interstitial region. This suggested that the cytoplasm location of the FN1 protein was stage‐specific, possibly occurring in the late stage of human cardiomyocyte hypertrophy, and this also served as a control supporting the specificity of the FN1 antibody. We did not replicate the expression patterns of FN1, CTGF and DCN in a knock‐in mouse HCM model, using the same antibodies and same conditions for the human sample experiments. This is a new HCM mouse model made by us, which has not been published. In 16 weeks after birth, the HCM mice exhibit clear cardiac hypertrophy and the Masson's trichrome staining demonstrates clear myocardial fibrosis of the heart tissue in the knock‐in mice. It should be noted that, both FN1 and CTGF were not elevated even in the interstitial region in this model. This may suggest that our findings are unique to human HCM, but more mouse models and disease stages should be examined to make this conclusion.

Together, by performing high‐resolution single‐cell RNA‐seq in individual cardiomyocytes obtained from the HCM patients and integrating with published HCM sc(sn)RNA‐seq dataset, our results comprehensively revealed the alterations of transcriptional networks in the hypertrophic cardiomyocytes that may provide valuable information for further research on the mechanisms of HCM.

## 4 MATERIALS AND METHODS

### 4.1 Human heart samples

This study was approved by the Ethics Committee of Fuwai Hospital, Chinese Academy of Medical Sciences. Written informed consent was obtained from each subject. Interventricular septum specimens were collected from 7 patients with obstructive HCM who underwent surgical myectomy and 2 control donors without cardiac diseases and not present left ventricular hypertrophy on echocardiography (Table S1). The HCM was diagnosed by the presence of maximum left ventricular wall thickness of ≥15 mm measured with echocardiography and/or cardiac magnetic resonance imaging after excluding other diseases that can cause such cardiac hypertrophy according to the 2014 guidelines of the European Society of Cardiology.^2^ After excised the fresh cardiac tissues were immediately subjected to cell isolation or paraffin‐embedded.

### 4.2 Human cardiomyocyte‐enriched digestion

Isolation of adult human cardiomyocytes was performed as follows. The whole isolation procedure was performed in a 37°C water bath in the presence of continuous oxygen flow (95% O_2_/5% CO_2_). In brief, samples were transported in prechilled University of Wisconsin (UW) solution, minced into small pieces, and transferred into an oxygenated Ca^2+^‐free solution. The samples were first digested with buffer containing 275 U/mL collagenase II, 1.2 U/mL protease, 1 mg/mL BSA and 0.015 mmol/L CaCl_2_, after which the samples were centrifuged and the supernatant was discarded. The subsequent digestion buffer was the same composition as described above but without protease. The digested cardiomyocytes were finally resuspended in Kraftbruhe (KB) solution and processed for single‐cell selection.

### 4.3 Single‐cell RNA‐seq library preparation and sequencing

Single‐cell RNA‐seq libraries were prepared by using a modified procedure based on the STRT‐seq protocol.^39^ After tissue digestion, individual cardiomyocytes were picked into 2 μL of cell lysis buffer under a microscope. Reverse transcription was performed with oligo dT primers composed of 8‐nt cell‐specific barcodes, 8‐nt unique molecular identifiers (UMIs) and 25‐nt oligo dT. Second‐strand cDNAs were synthesized and subjected to 19 cycles of PCR amplification with the 3′ P2 primer and the IS primer. Then, 96 different barcoded, pre‐amplified single‐cell PCR products were pooled together and purified with AMPure XP beads (Beckman). Forty nanograms of DNA were then subject to four cycles of PCR with the IS primer and biotin‐modified index primer. Index‐induced cDNA was sheared to ~300 bp fragments on a Covaris S2. Fragmented DNA was then enriched by incubating the solutions with streptavidin C1 beads (Thermo Fisher) for 1 h. Finally, the libraries were constructed using a KAPA Hyper Prep Kit (KAPA Biosystems). All single‐cell RNA‐seq data were generated on an Illumina HiSeq4000 platform for 150‐bp paired‐end reads.

### 4.4 Immunofluorescence staining

We applied an immunofluorescence staining assay to validate specific gene expression in our single‐cell RNA‐seq data. Five micrometres sections were obtained with Lecia RM2235 for paraffin‐embedded heart tissues. After paraffin sections were dewaxed with gradient ethanol, they were subjected to antigen retrieval using a pressure cooker in sodium citrate buffer (PH 6.0), and incubated in a blocking solution (3% Bovine Serum Albumin, 1% normal Goat serum, 0.1% Triton X‐100 in PBS), followed by incubation with the primary antibodies overnight at 4°C. Cell nuclei were stained with DAPI. Immunofluorescence images were acquired through Leica SP8 confocal microscope (Leica Microsystems) with an optical section of 1.271 μm. For quantitative analysis, ImageJ was used to calculate the mean fluorescence intensity. The detailed information of the antibodies and experimental conditions were list in Table S8.

### 4.5 Processing of single‐cell RNA‐seq data

Cell barcodes and UMI sequences in read 2 were first extracted by Umi_tools (v1.0.0)^40^ and attached after the read name of read 1. TSO and polyA sequences were removed by local scripts and then mapped to the hg38 human genome using STAR (2.7.2b).^41^ After gene features were assigned by featureCounts (v2.0.0),^42^ the final UMI count expression matrix was generated by the ‘count’ function of UMI tools, which were normalized to TPM (Transcripts per million), transformed to log(TPM/10 + 1) and scaled using Seurat(v3.1.3)^43^ procedure.

### 4.6 Quality control of single‐cell RNA‐seq data

We applied two‐step filter criteria to all the cell samples. First, we excluded cells that expressed fewer than 2000 genes or had a mitochondrial read fraction greater than 20% (Figure S1a). Second, we removed potentially contaminated cell clusters by clustering the remaining HCM and normal cardiomyocytes after batch effect correction (Figure S1b–d). Differential expression analysis identified a cluster up‐regulating many non‐cardiomyocyte marker genes (including *FABP5*, *PECAM1*, *VWF* and *ENG*) while having fewer down‐regulated genes (Figure S1e,f), which also enriched in GO terms such as Blood vessel development (Figure S1g). Thus, we identified this cluster as potentially contaminated cells and removed them from further analysis.

### 4.7 Cell clustering and batch correction

After obtaining cells that passed the quality control, we first used the R package Seurat (v3.1.3)^43^ to perform PCA (Principal Component Analysis) and UMAP (Uniform Manifold Approximation and Projection) dimension reduction and find cell clusters by a shared‐nearest‐neighbour (SNN) modularity optimization‐based clustering algorithm. We used Harmony (v1.0)^44^ to remove batch effects.

### 4.8 Differential expression analysis

We used the Seurat^43^, ^45^ function ‘FindMarkers’ with parameters ‘min.pct = 0.25, logfc.threshold = 0.5’ to find differentially expressed genes (DEGs) between HCM patients and normal control. Differential expression analysis during quality control was performed using the ‘FindAllMarkers’ function with the same parameters as mentioned above, and only DEGs with an adjusted *p*‐value < 0.05 were included.

### 4.9 Enrichment analysis and GSEA analysis

DEGs with adjusted *p*‐value < 0.05 and log‐scaled fold change > 0.5 were used to perform enrichment analysis online at Metascape^46^ (http://metascape.org). We used the ‘GSEA’ function in R package clusterProfiler (v3.14.3)^47^ to perform Gene Set Enrichment Analysis (GSEA) analysis in Kyoto Encyclopedia of Genes and Genomes (KEGG) pathway genes sets.

### 4.10 Single‐cell regulatory network inference and clustering (SCENIC) analysis

SCNEIC was performed with pySCENIC,^20^ which identified activated transcription factors (TFs) and related regulatory networks of each TF (named regulon). Then the AUC matrix for each cell and regulon output from SCENIC were used as input to perform PCA, and the top 30 components of PCA were further used for t‐distributed stochastic neighbour embedding (TSNE) dimensional reduction.

### 4.11 Gene module score analysis

Module scores of given gene sets for every single cell were calculated by the ‘AddModuleScore’ function of the Seurat package with default parameters, which calculate the average expression levels of each gene set on then subtracted by the expression of control gene sets.

### 4.12 Analysis of co‐expression gene module

To reveal the co‐expression relation between genes and find the gene module, Pearson's correlation coefficients for each pair of genes in all single‐cell were calculated, resulting in a correlation matrix, which was used to perform hierarchical clustering analysis and generate clustered heatmap by‘pheatmap’ function of ‘pheatmap’ package with parameter ‘cluster.method = ‘ward.D2”.

### 4.13 Ligand‐receptor analysis

CellPhoneDB (v2.1.4)^48^ was used to conduct the ligand‐receptor analysis. Only the interacting partners with a *p*‐value < 0.01 and mean expression values > 1 were included.

## AUTHOR CONTRIBUTIONS

Lei Song, Lu Wen, Shuiyun Wang and Jizheng Wang conceived the project. Jiansen Lu, Jie Ren, Jie Liu, Yueli Cui, Yuhan Liao, Yuan Zhou, Yun Gao and Fuchou Tang performed the scRNA‐seq experiments. Minjie Lu performed the immunofluorescence staining. Jiansen Lu conducted the bioinformatics analyses. Jiansen Lu, Jie Ren, Jie Liu, Lei Song, Lu Wen, Shuiyun Wang and Jizheng Wang wrote the manuscript with help from all of the authors. All authors read and approved the final manuscript.

## FUNDING INFORMATION

This work was supported by the National Key R&D Program of China (2017YFA0103402), CAMS Innovation Fund for Medical Sciences (2023‐I2M‐1‐001), the Science and Technology Department of Heilongjiang Province (2022ZXJ03C04), the National Natural Science Foundation of China (81870286) and the Open Research Fund of the National Center for Protein Sciences at Peking University in Beijing.

## CONFLICT OF INTEREST STATEMENT

The authors declare no competing financial interests.

## CODE AVAILABILITY

The scripts associated with this research are available from the corresponding authors upon reasonable request.

## INFORMED CONSENT

Written informed consent was obtained from all subjects or parents.

## Supporting information


**Figure S1.** Quality control and removal of potential contaminants. (a) Mapping rate (left), number of detected transcripts, genes and percentage of mitochondrial counts (right) in each library. (b) PCA and UMAP visualization of all cells from HCM and NC, a strong batch effect could be observed. (c) UMAP after correction of batch effect by Harmony, cells were coloured by the source of patients (left) and cell clusters (right), respectively. (d) Heatmap of DEGs between two clusters in (c), indicating up‐regulated of non‐cardiomyocyte genes in the filtered cluster. (e, f) Expression of representative endothelial marker genes, visualized with feature plot (e) and violin plot (f), respectively. (g) Gene ontology enrichment result of filtered cluster up‐regulated DEGs.
**Figure S2.** Gene correlation analysis for each individual. (a–d) Pearson's correlation of *NPPA‐NPPB*, *NPPB‐TPM3*, *NPPB‐ACE2* and *LUM‐DCN* in each individual.
**Figure S3.** Expression of representative genes in cardiomyocytes of HCM and NC. (a, b) Violin plot showing the expression of previously identified causal genes related to HCM. (c, d) Violin plot showing the expression of metallothionein genes, Wilcoxon rank sum test was used to determine *p*‐value (see also in Methods). ***p‐value < 0.001.
**Figure S4.** Single‐cell regulatory network inference and clustering. (a) PCA visualization with SCENIC AUC scores of regulons. (b) Representative down‐regulated differential activated regulons and the expression of their target genes in HCM and NC. (c) Regulon activities and the expression of their corresponding transcription factors. (d) Venn diagram showing the overlapping of differentially activated regulons (DARs) with differential expressional transcriptional factors (DETFs) in HCM and NC.
**Figure S5.** Low depth of RNA sequencing reduces the sensitivity in the detection of DEGs. (a) Pie chart and table showed the up‐regulated genes in this study (adjusted *p*‐value < 0.05; fold change > 1.5) shared by other human single‐cell or single‐nucleus datasets. Genes were classified into different DEG groups. (b) Normalized expression levels of up‐regulated genes of this study in each dataset. (c) The violin plot represents the normalized expression levels of DEG groups in each dataset. (d) The pair‐wise comparison result of the Tukey HSD test for the normalized expression of the DEGs group in each dataset. N.S.: not significant; *p‐value < 0.05; **p‐value < 0.01; ***p‐value < 0.001.
**Figure S6.** Gene modules of signature genes revealed by co‐expression analysis. (a) Co‐expression heatmap of signature up‐regulated genes in cardiomyocytes from HCM from three datasets, visualized by Pearson's correlation coefficient between genes. Gene modules were classified by unsupervised hierarchical clustering. (b) The Sankey plot showed the correspondence relationships among the up‐regulated gene modules of four datasets. (c) Venn diagram and upset plot of down‐regulated differential expressed genes identified from four datasets (adjuster *p*‐value < 0.05, fold change > 1.5), respectively. (d) Co‐expression heatmap of signature up‐regulated genes in cardiomyocytes from HCM from the four datasets, visualized by Pearson's correlation coefficient between genes. Gene modules were classified by unsupervised hierarchical clustering. (e) The Sankey plot showed the correspondence relationships among the down‐regulated gene modules of four datasets.
**Figure S7.** Gene ontology enrichments of gene modules of signature regulated genes.
**Figure S8.** Gene ontology enrichments of gene modules of signature regulated genes. (a) Volcano plots showing DEGs between wildtype and experimental groups in previous studies. *NPPA*, *NPPB*, as well as ECM genes, were highlighted. (b) The expression level of myocardial ECM genes among different experimental groups in previous and current studies. Sham: Sham‐operated control mice; TAC: transverse aortic constriction mouse model of cardiac hypertrophy; WT: wild‐type; Saline and AngII: mice continuously infused with saline (Saline, control) or angiotensin II (AngII, cardiac hypertrophy model), respectively, for 2 weeks; DCM: dilated cardiomyopathy. (c) Venn diagram and upset plot showing the overlapping of up‐regulated DEGs among Nomura et al., See et al. and current study. (d) The overlapping DEGs among these datasets, ECM genes were highlighted in red.
**Figure S9.** Expression of ECM genes in HCM datasets. (a, b) The violin plot showed the module score of the ‘extracellular matrix organization’ pathway (R‐HAS‐1474244) in our data (A) and other human HCM datasets. (c) Heatmap showed the differential expressed genes between HCM and NC belonging to the ‘extracellular matrix organization’ pathway. (d) Expression levels of representative ECM genes in four HCM datasets, coloured by average expression levels. The size of the dot represented the percentage of expressed cells.
**Figure S10.** Immunofluorescence staining of ECM genes in HCM and NC. (a, b) Immunofluorescence staining of DCN and VIM in HCM and NC. Individual cardiomyocytes were defined using CTNT (green) and DAPI (blue). Individual cardiomyocytes were defined using *CTNT* (green) and DAPI (blue). *DCN* and *VIM* are ECM genes (red). Scale bar, 50 μm.
**Figure S11.** Region of interest (ROI) regions used for quantitative analysis. Representative immunofluorescence staining images and related region of interest (ROI) regions were used for quantitative analysis. Each gene was replicated in three patients and three normal controls.
**Figure S12.** Immunofluorescence staining of representative ECM genes in HCM and wildtype mouse. A double knock‐in HCM mouse model was used in this study (unpublished), which was created by introducing human disease‐causing mutations in *Myh6* and *Tnnt2*. The 16 weeks old wildtype and HCM mice were used for experiment. (a, b) Masson's trichrome staining of heart transections showed the myocardial fibrosis in double mutant knock‐in HCM model (a) and wildtype mouse (b). Scale bars were 1 mm and 100 μm in the upper and lower panels, respectively. (c, d) Immunofluorescence staining of representative ECM genes. Individual cardiomyocytes were defined using CTNT (green) and DAPI (blue). ECM related genes including CTGF (c) and FN1 (d) were coloured in red. Scale bar, 50 μm.
**Figure 13.** Analysis of bulk RNA‐seq data from MAGNet. (a) PCA visualizations of all MAGNet bulk RNA samples. (b) Normalized expression levels of *NPPA*, *NPPB*, *ACTN1* and *ACTA1* in each group of the MAGNet dataset. (c) Module score of signature up‐ and down‐regulated genes in MAGNet dataset. (d) Correlation between signature up‐ and down‐regulated gene module score and PC2 values of each sample. (e) Relative gene module scores of signature gene modules in MAGNet dataset. Each dot was coloured by the relative module score between HCM and NC, the size of the dot represented the −log_10_(*p*‐value), two‐sided *t*‐test was used to determine the significance levels. (f) Expression of *TCF4*, *ETV1*, *KLF5*, *CEBPB* and *CEBPD* in the MAGNet dataset, coloured by average expression levels. The size of the dot represented the percentage of expressed cells. (g) Relative gene module scores of target gene regulons of *TCF4*, *ETV1*, *KLF5*, *CEBPB* and *CEBPD* in the MAGNet dataset. Each dot was coloured by the relative module score compared with NC group, the size of the dot represented the −log_10_(*p*‐value), two‐sided *t*‐test was used to determine the significance levels.

**Table S1.** Information of patients.

**Table S2.** Differentially expressed genes between cardiomyocytes of HCM and NC.

**Table S3.** Ligand and receptor pairs were identified in cardiomyocytes of HCM and NC.

**Table S4.** Gene ontology enrichment results of DEGs between HCM and NC.

**Table S5.** Signature up‐ and down‐regulated genes revealed by comparative analysis.

**Table S6.** Gene modules of signature up‐ and down‐regulated genes.

**Table S7.** Differentially expressed genes between subpopulations of HCM cardiomyocytes.

**Table S8.** Antibody information for immunostaining.

## Data Availability

The raw data are deposited in the Genome Sequence Archive (GSA) databases with accession number HRA000328. We also created an interactive website for this study (https://tanglab.shinyapps.io/HCM_scRNA/). All other data supporting the findings of this study are available from the corresponding authors upon reasonable request.
