# Supplementary material for: High‐resolution single‐cell transcriptomic survey of cardiomyocytes from patients with hypertrophic cardiomyopathy
Source: Cell Prolif. 2023 Sep 28;57(3):e13557. doi: 10.1111/cpr.13557 (PMC10905351; doi:10.1111/cpr.13557)
Supplement: Supplementary file 1 — Figure S1. Quality control and removal of potential contaminants. (a) Mapping rate (left), number of detected transcripts, genes and percentage of mitochondrial counts (right) in each library. (b) PCA and UMAP visualization of all cells from HCM and NC, a strong batch effect could be observed. (c) UMAP after correction of batch effect by Harmony, cells were coloured by the source of patients (left) and cell clusters (right), respectively. (d) Heatmap of DEGs between two clusters in (c), indicating up‐regulated of non‐cardiomyocyte genes in the filtered cluster. (e, f) Expression of representative endothelial marker genes, visualized with feature plot (e) and violin plot (f), respectively. (g) Gene ontology enrichment result of filtered cluster up‐regulated DEGs. Figure S2. Gene correlation analysis for each individual. (a–d) Pearson's correlation of NPPA‐NPPB, NPPB‐TPM3, NPPB‐ACE2 and LUM‐DCN in each individual. Figure S3. Expression of representative genes in cardiomyocytes of HCM and NC. (a, b) Violin plot showing the expression of previously identified causal genes related to HCM. (c, d) Violin plot showing the expression of metallothionein genes, Wilcoxon rank sum test was used to determine p‐value (see also in Methods). ***p‐value < 0.001. Figure S4. Single‐cell regulatory network inference and clustering. (a) PCA visualization with SCENIC AUC scores of regulons. (b) Representative down‐regulated differential activated regulons and the expression of their target genes in HCM and NC. (c) Regulon activities and the expression of their corresponding transcription factors. (d) Venn diagram showing the overlapping of differentially activated regulons (DARs) with differential expressional transcriptional factors (DETFs) in HCM and NC. Figure S5. Low depth of RNA sequencing reduces the sensitivity in the detection of DEGs. (a) Pie chart and table showed the up‐regulated genes in this study (adjusted p‐value < 0.05; fold change > 1.5) shared by other human single‐cell [file CPR-57-e13557-s006.docx]

**Supplemental Figures**

**
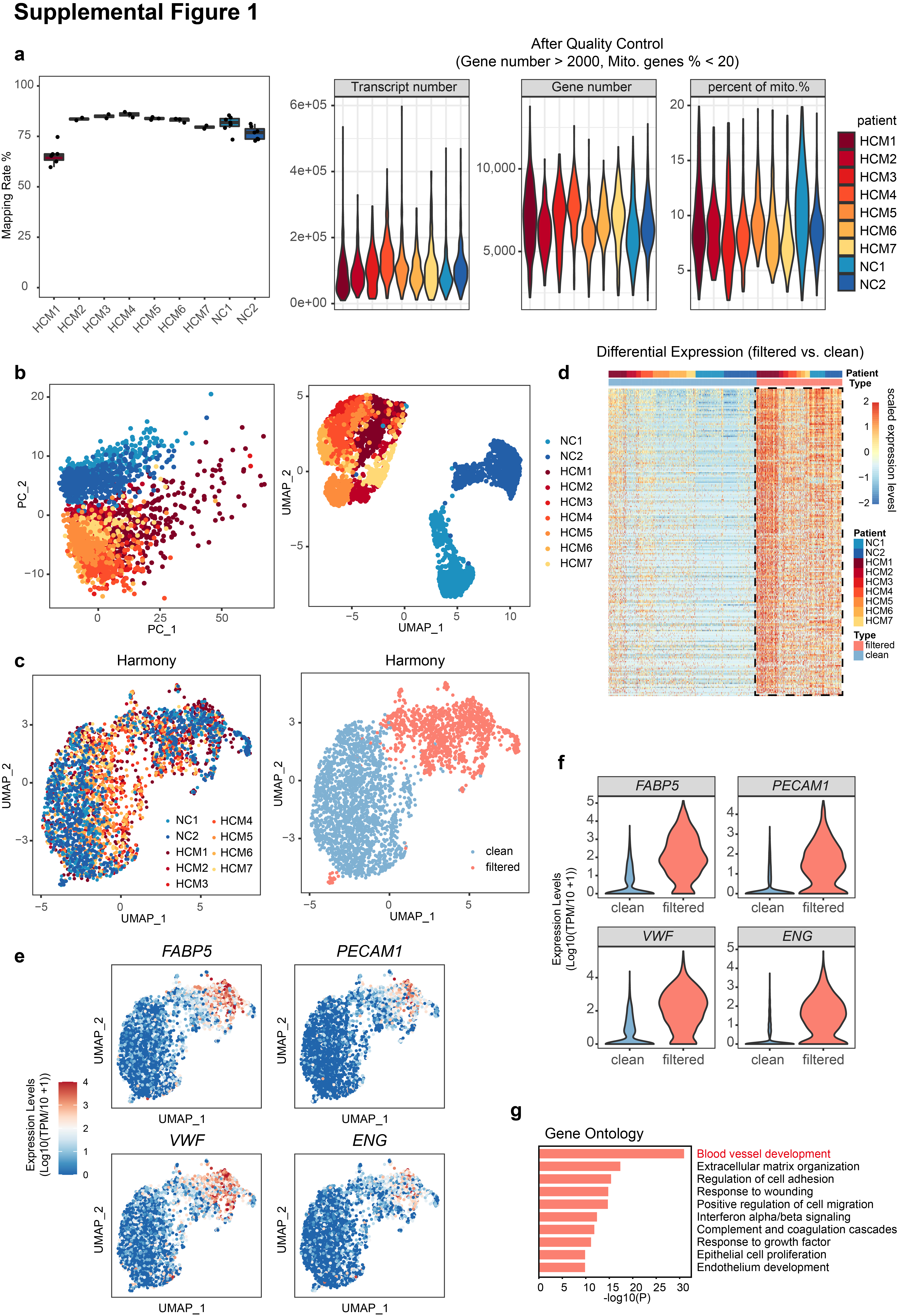
**

**Supplemental Figure 1. Quality control and removal of potential contaminants.**

**a.** Mapping rate (left), number of detected transcripts, genes, and percentage of mitochondrial counts (right) in each library. **b.** PCA and UMAP visualization of all cells from HCM and NC, a strong batch effect could be observed. **c.** UMAP after correction of batch effect by Harmony, cells were colored by the source of patients (left) and cell clusters (right), respectively. **d.** Heatmap of DEGs between two clusters in (**c**), indicating upregulated of non-cardiomyocyte genes in the filtered cluster. **e-f.** Expression of representative endothelial marker genes, visualized with feature plot (**e**) and violin plot (**f**), respectively. **g.** Gene Ontology enrichment result of filtered cluster upregulated DEGs.

**
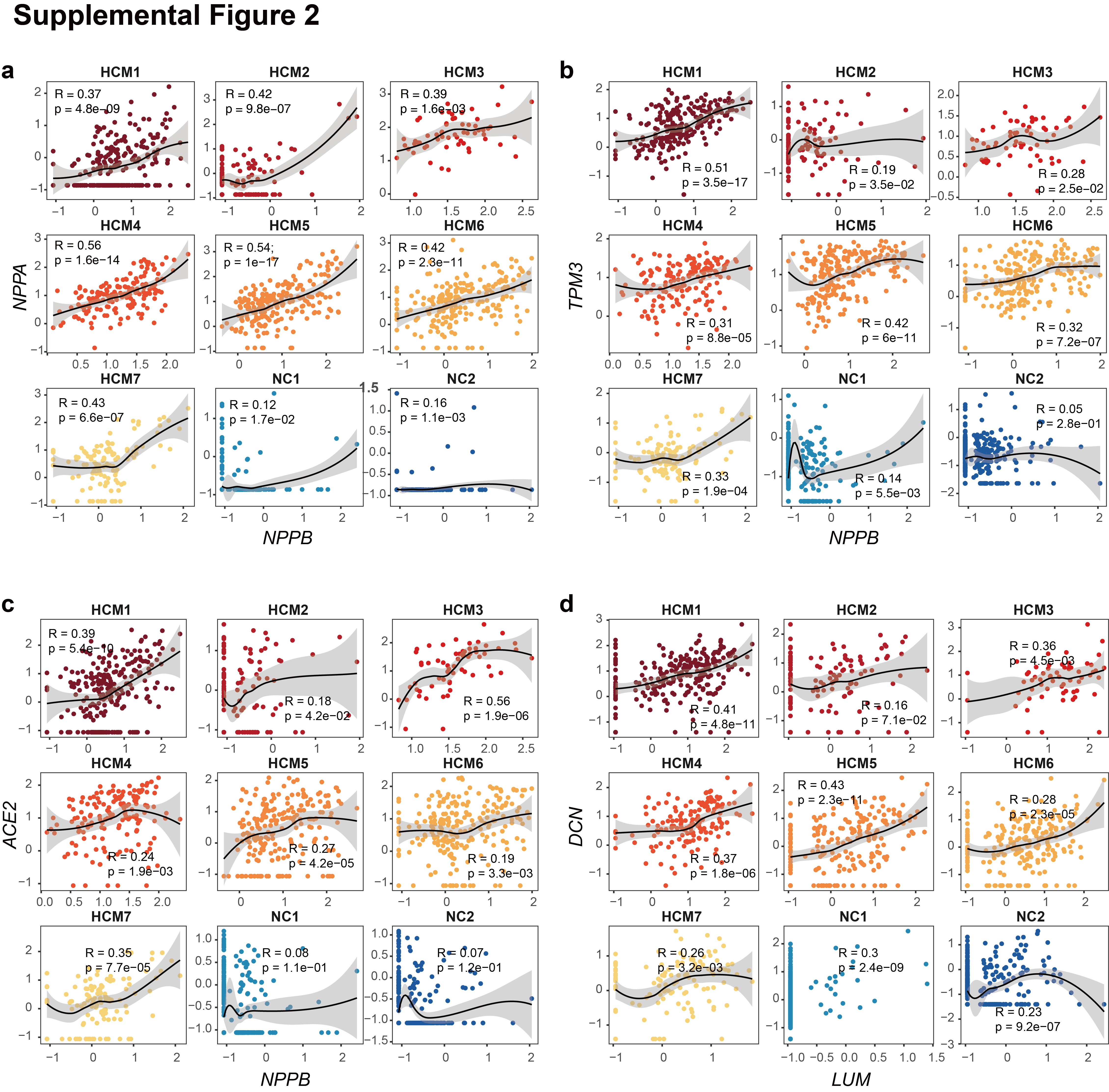
**

**Supplemental Figure 2. Gene correlation analysis for each individual.**

**a-d.** Pearson’s correlation of *NPPA-NPPB*, *NPPB-TPM3*, *NPPB-ACE2,* and *LUM-DCN* in each individual.

**
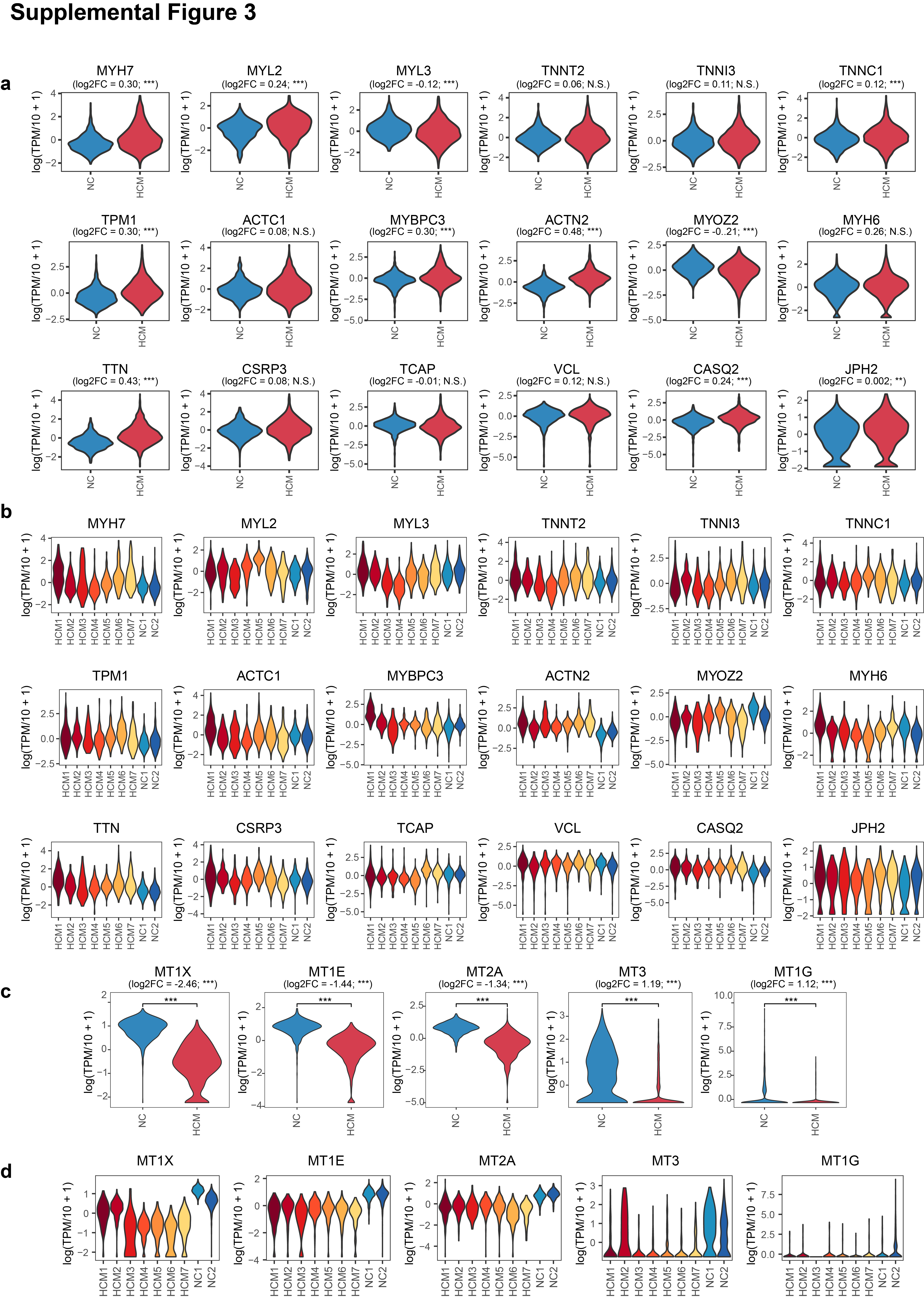
**

**Supplemental Figure 3. Expression of representative genes in cardiomyocytes of HCM and NC.**

**a-b.** Violin plot showing the expression of previously identified causal genes related to HCM. **c-d.** Violin plot showing the expression of metallothionein genes, Wilcoxon rank sum test was used to determine *P*-value (see also in Method). *** *P*-value < 0.001.

**
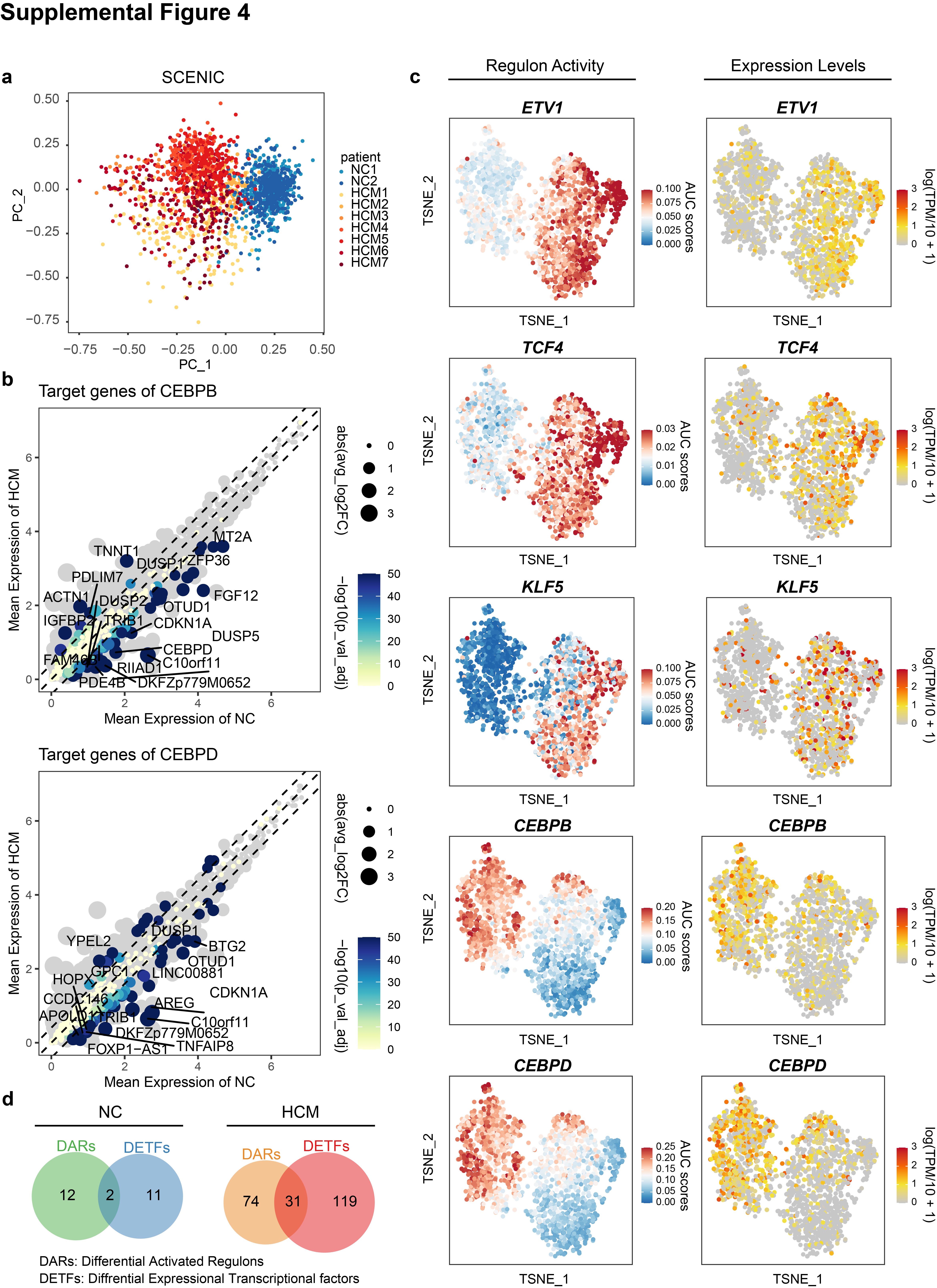
**

**Supplemental Figure 4. Single-cell regulatory network inference and clustering.**

**a.** PCA visualization with SCENIC AUC scores of regulons. **b.** representative downregulated differential activated regulons and the expression of their target genes in HCM and NC. **c.** Regulon activities and the expression of their corresponding transcription factors. **d.** Venn diagram showing the overlapping of differentially activated regulons (DARs) with differential expressional transcriptional factors (DETFs) in HCM and NC.

**
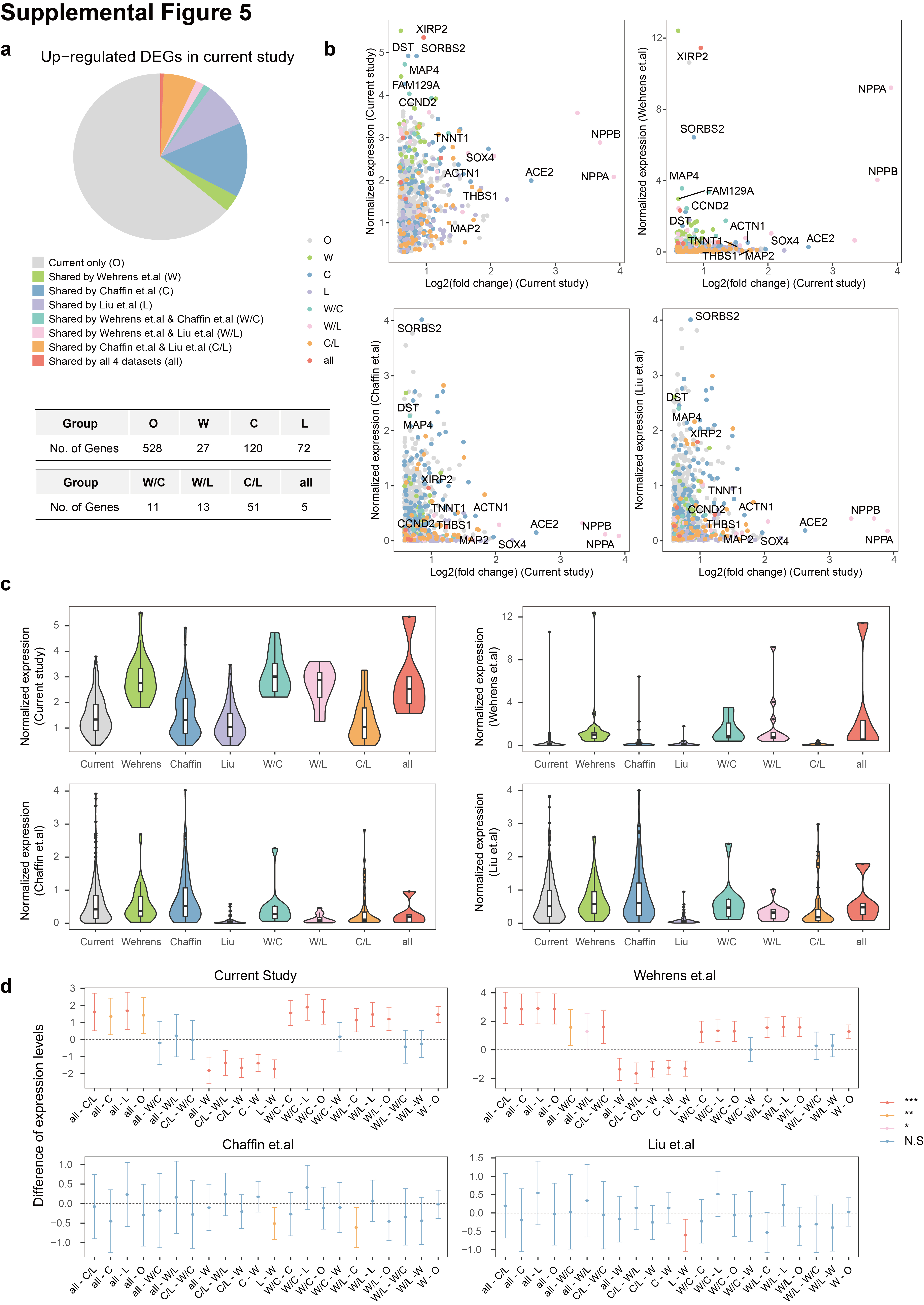
**

**Supplemental Figure 5. Low depth of RNA sequencing reduces the sensitivity in the detection of DEGs.**

**a.** Pie chart and table showed the up-regulated genes in this study (adjusted p-value < 0.05; fold change > 1.5) shared by other human single-cell or single-nucleus datasets. Genes were classified into different DEG groups. **b.** Normalized expression levels of up-regulated genes of this study in each dataset. **c.** The violin plot represents the normalized expression levels of DEG groups in each dataset. **d.** The pair-wise comparison result of the Tukey HSD test for the normalized expression of the DEGs group in each dataset. N.S. not significant; * *P*-value < 0.05; ** *P*-value < 0.01; *** *P*-value < 0.001.

**
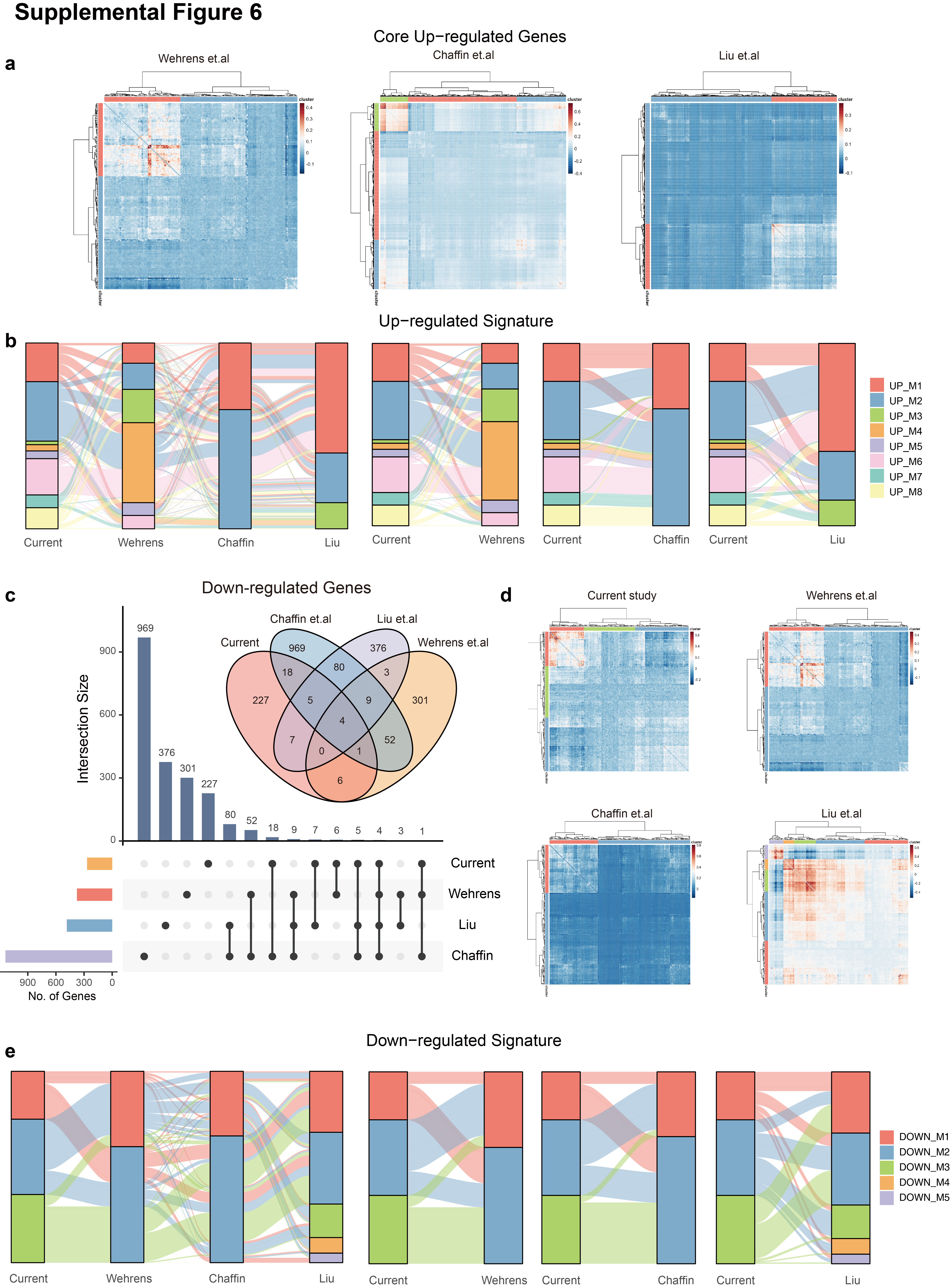
**

**Supplemental Figure 6. Gene modules of signature genes revealed by co-expression analysis.**

**a.** Co-expression heatmap of signature up-regulated genes in cardiomyocytes from HCM from three datasets, visualized by Pearson’s correlation coefficient between genes. Gene modules were classified by unsupervised hierarchical clustering. **b.** The Sankey plot showed the correspondence relationships among the up-regulated gene modules of four datasets. **c.** Venn diagram and upset plot of down-regulated differential expressed genes identified from four datasets (adjuster *P*-value < 0.05, fold change > 1.5), respectively. **d.** Co-expression heatmap of signature up-regulated genes in cardiomyocytes from HCM from the four datasets, visualized by Pearson’s correlation coefficient between genes. Gene modules were classified by unsupervised hierarchical clustering. **e.** The Sankey plot showed the correspondence relationships among the down-regulated gene modules of four datasets.

**
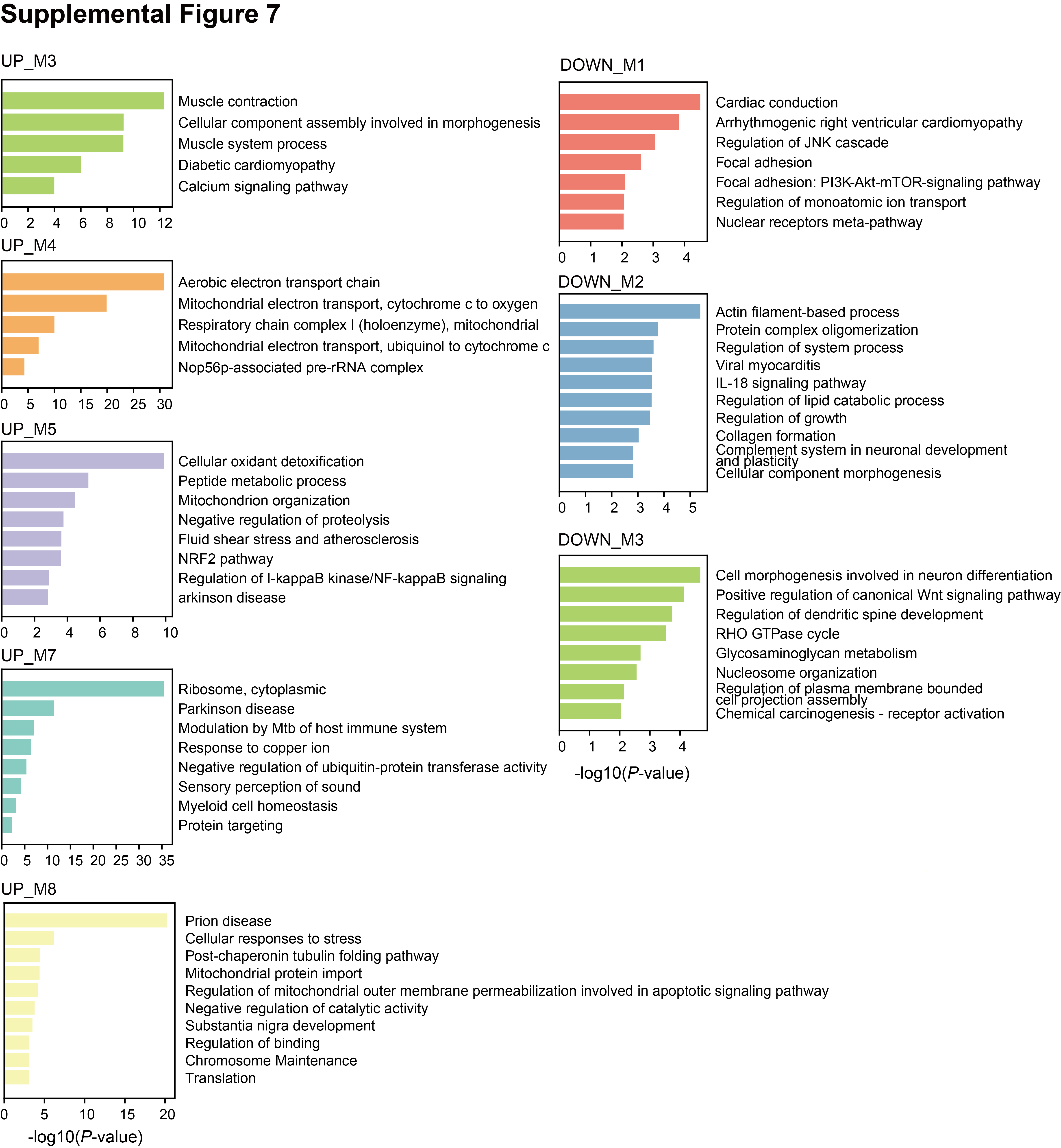
**

**Supplemental Figure 7. Gene ontology enrichments of gene modules of signature regulated genes.**

**
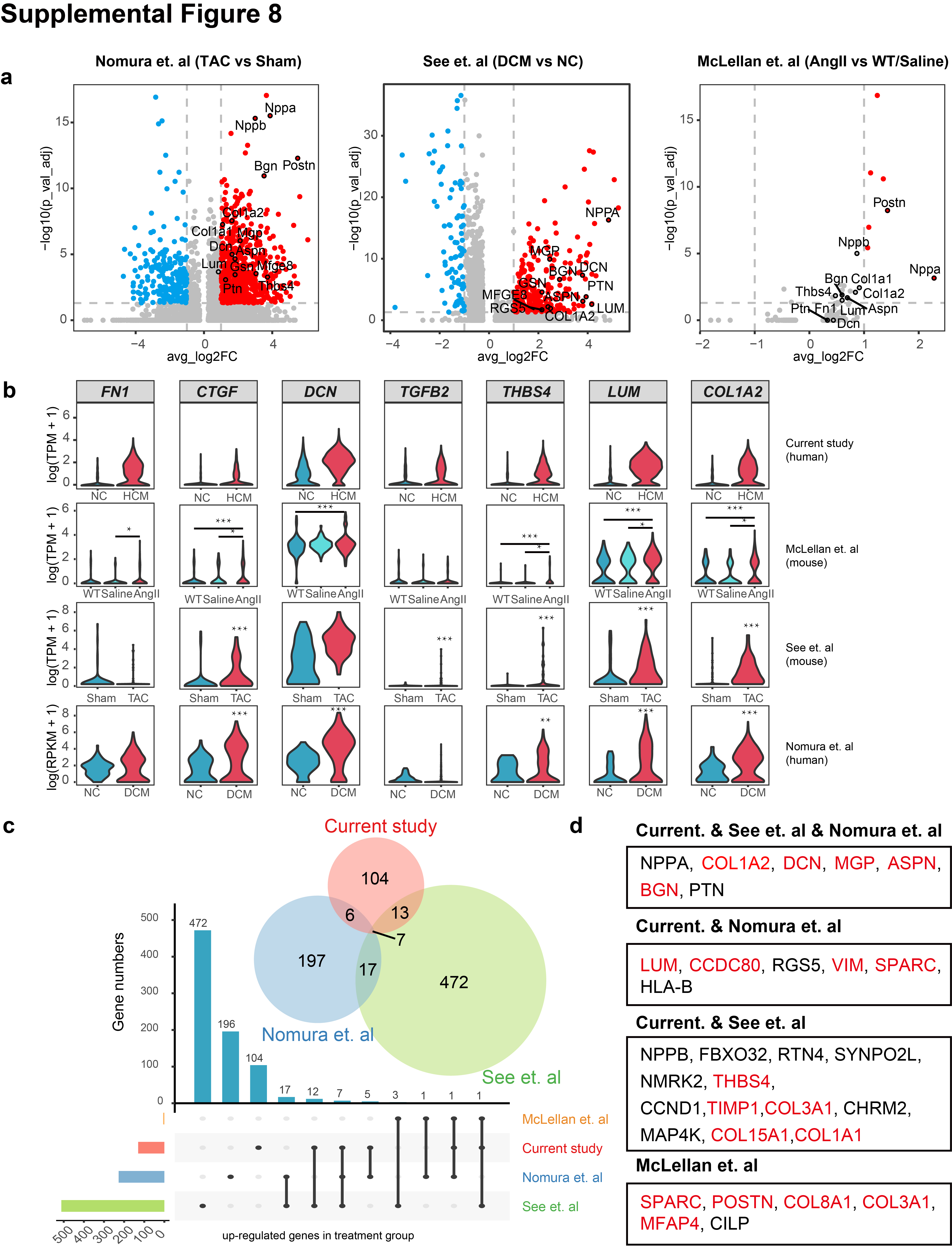
**

**Supplemental Figure 8. Gene ontology enrichments of gene modules of signature regulated genes.**

**a.** Volcano plots showing DEGs between wildtype and experimental groups in previous studies. *NPPA,* *NPPB*, as well as ECM genes, were highlighted. **b.** The expression level of myocardial ECM genes among different experimental groups in previous and current studies. Sham: Sham-operated control mice; TAC: transverse aortic constriction mouse model of cardiac hypertrophy; WT: wild-type; Saline and AngII: mice continuously infused with saline (Saline, control) or angiotensin II (AngII, cardiac hypertrophy model), respectively, for two weeks; DCM: dilated cardiomyopathy. **c.** Venn diagram and Upset plot showing the overlapping of upregulated DEGs among Nomura et.al, See et al and current study. **d.** The overlapping DEGs among these datasets, ECM genes
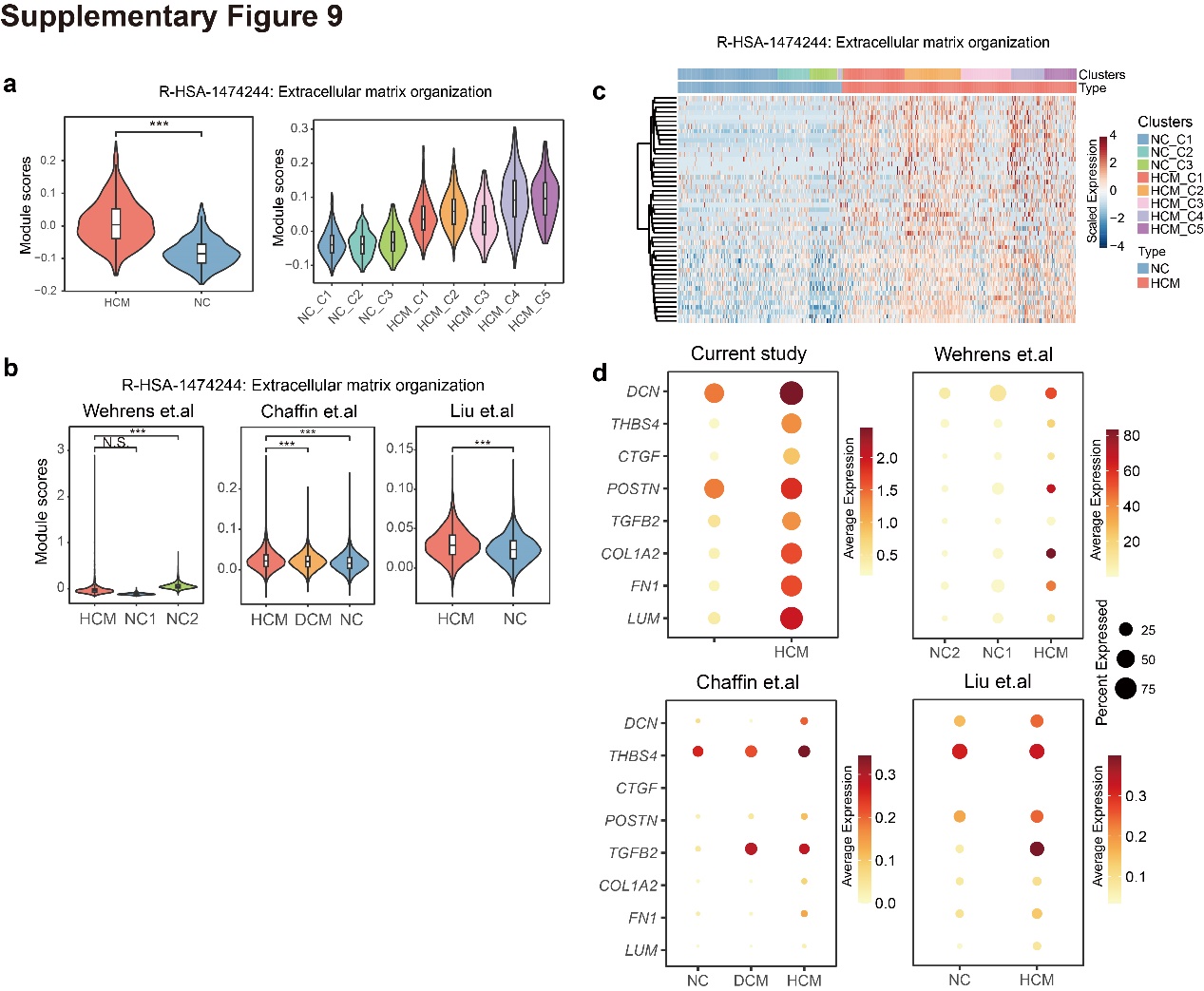
were highlighted in red.

**Supplemental Figure 9. Expression of ECM genes in HCM datasets.**

**a-b.** The violin plot showed the module score of the ‘extracellular matrix organization’ pathway (R-HAS-1474244) in our data (A) and other human HCM datasets. **c.** Heatmap showed the differential expressed genes between HCM and NC belonging to the ‘extracellular matrix organization’ pathway. **d.** Expression levels of representative ECM genes in four HCM datasets, colored by average expression levels. The size of the dot represented the percentage of expressed cells.

**
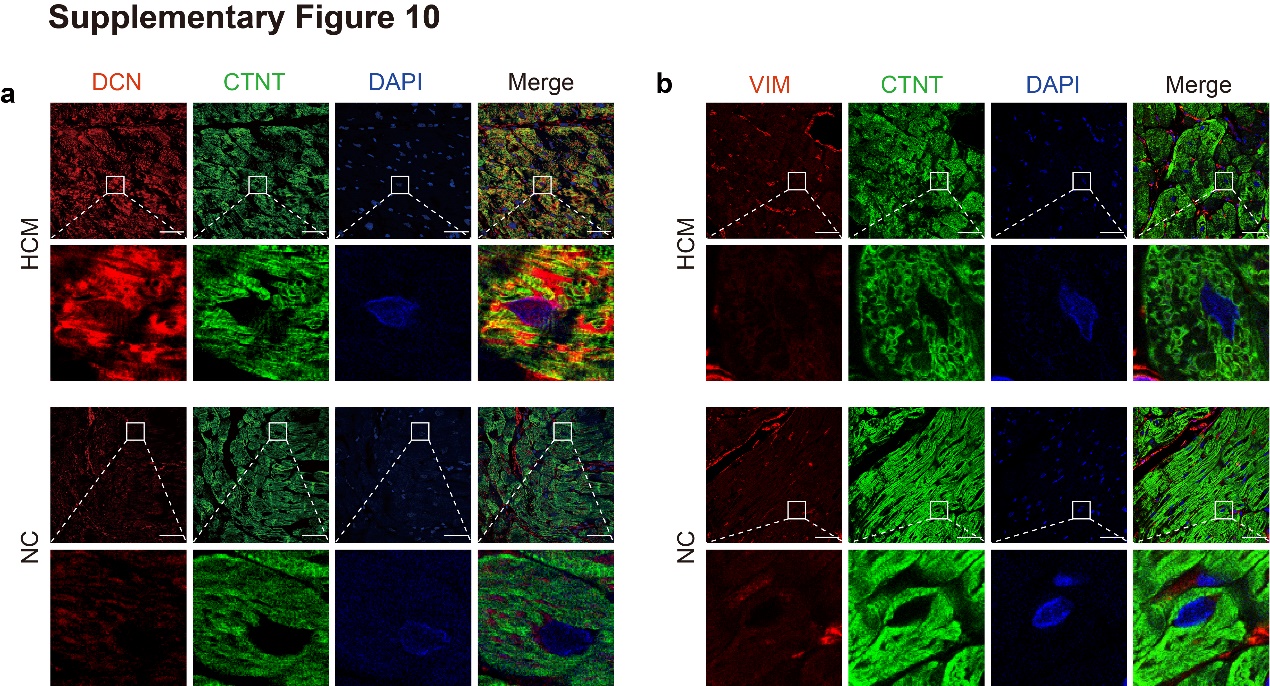
**

**Supplemental Figure 10. Immunofluorescence staining of ECM genes in HCM and NC.**

**a-b.** Immunofluorescence staining of DCN and VIM in HCM and NC. Individual cardiomyocytes were defined using CTNT (green) and DAPI (blue). Individual cardiomyocytes were defined using *CTNT* (green) and DAPI (blue). *DCN* and *VIM* are ECM genes (red). Scale bar, 50 μm.


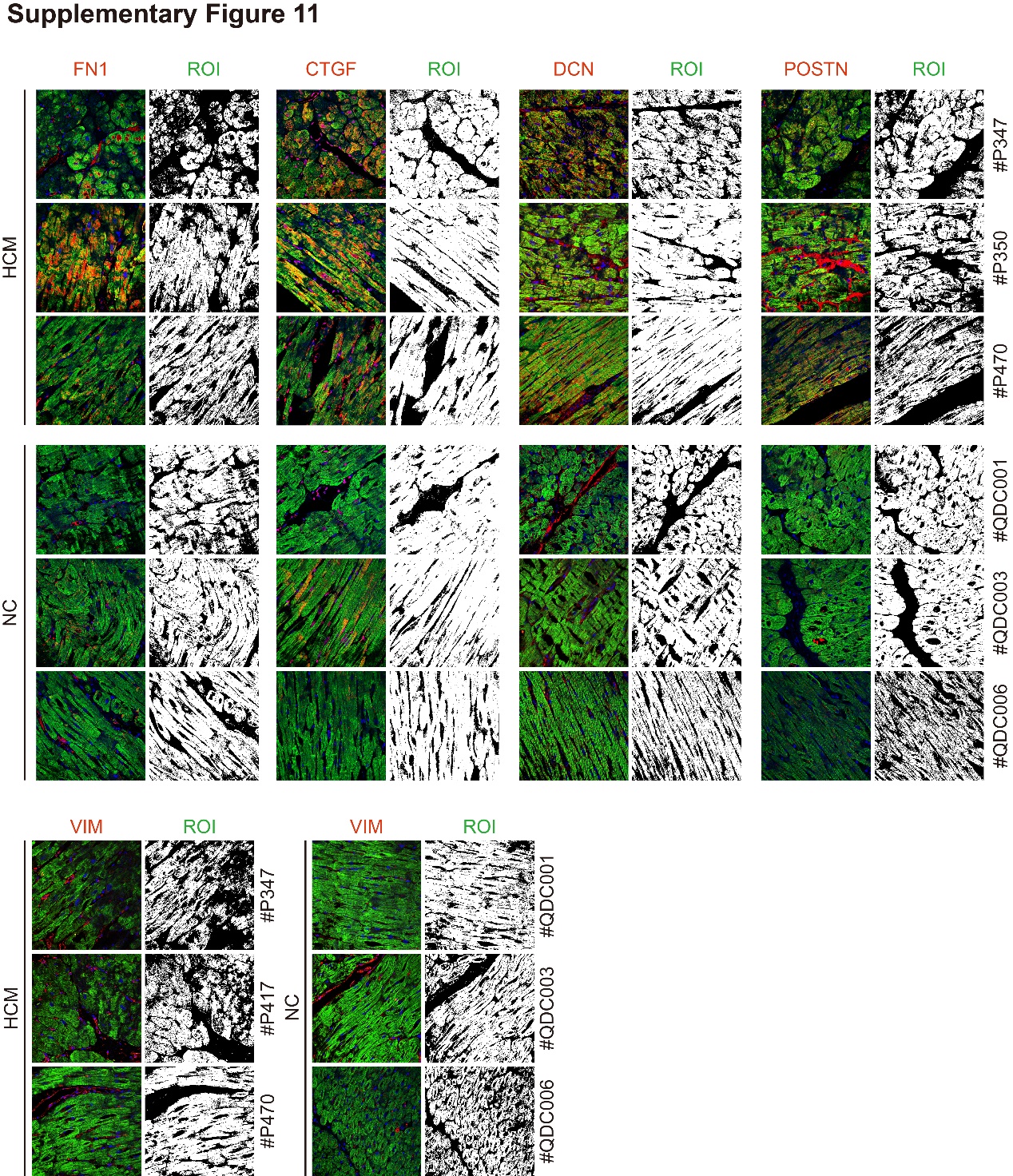


**Supplemental Figure 11. Region of interest (ROI) regions used for quantitative analysis.**

Representative immunofluorescence staining images and related region of interest (ROI) regions were used for quantitative analysis. Each gene was replicated in three patients and three normal controls.

**
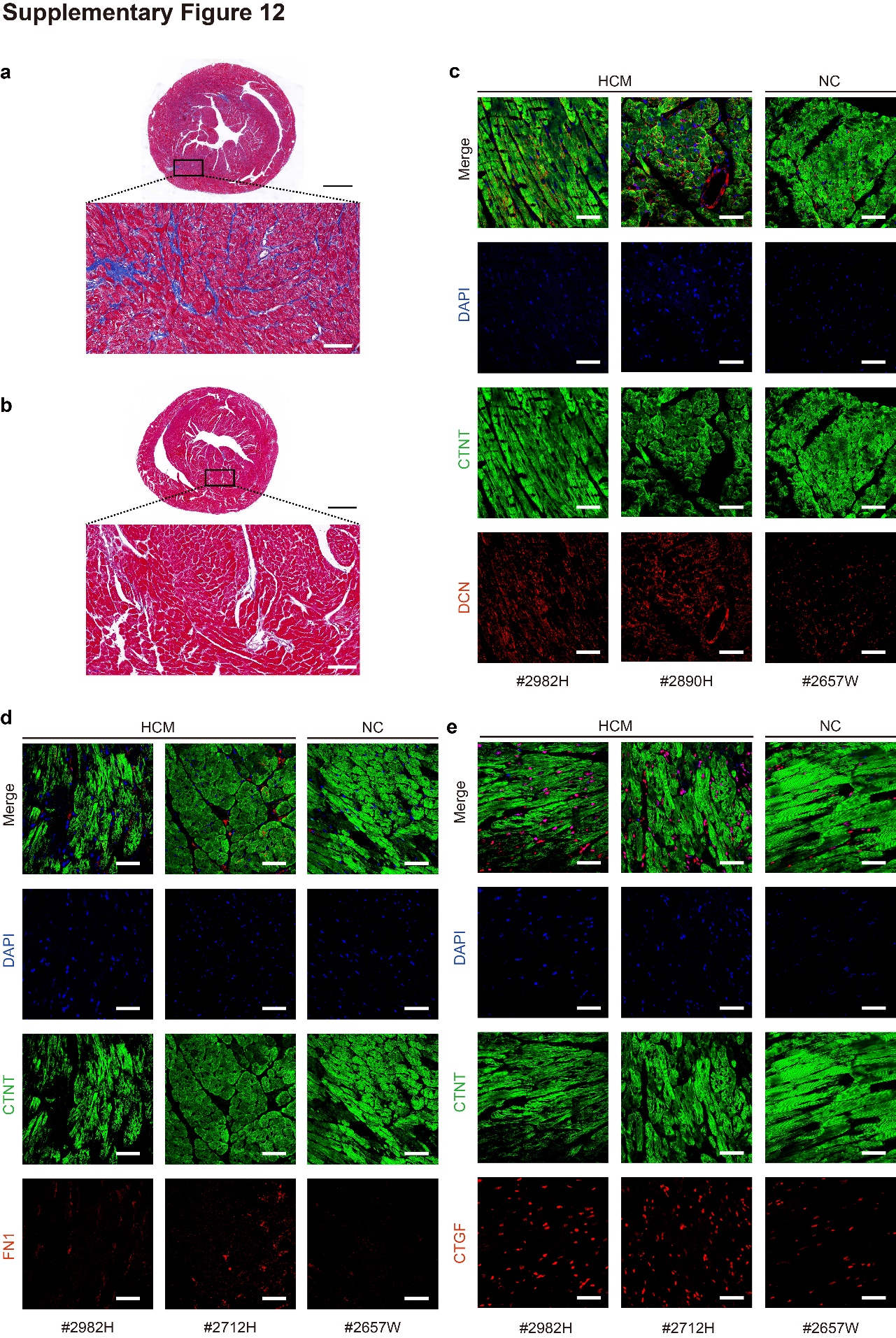
**

**Supplemental Figure 12. Immunofluorescence staining of representative ECM genes in HCM and wildtype mouse.**

A double knock-in HCM mouse model was used in this study (unpublished), which was created by introducing human disease-causing mutations in *Myh6* and *Tnnt2*. The 16 weeks old wildtype and HCM mice were used for experiment. **a-b.** Masson’s trichrome staining of heart transections showed the myocardial fibrosis in double mutant knock-in HCM model (**a**) and wildtype mouse (**b**). Scale bars were 1mm and 100 μm in the upper and lower panels, respectively. c-d. Immunofluorescence staining of representative ECM genes. Individual cardiomyocytes were defined using CTNT (green) and DAPI (blue). ECM related genes including CTGF (**c**) and FN1 (**d**) were colored in red. Scale bar, 50 μm.

**
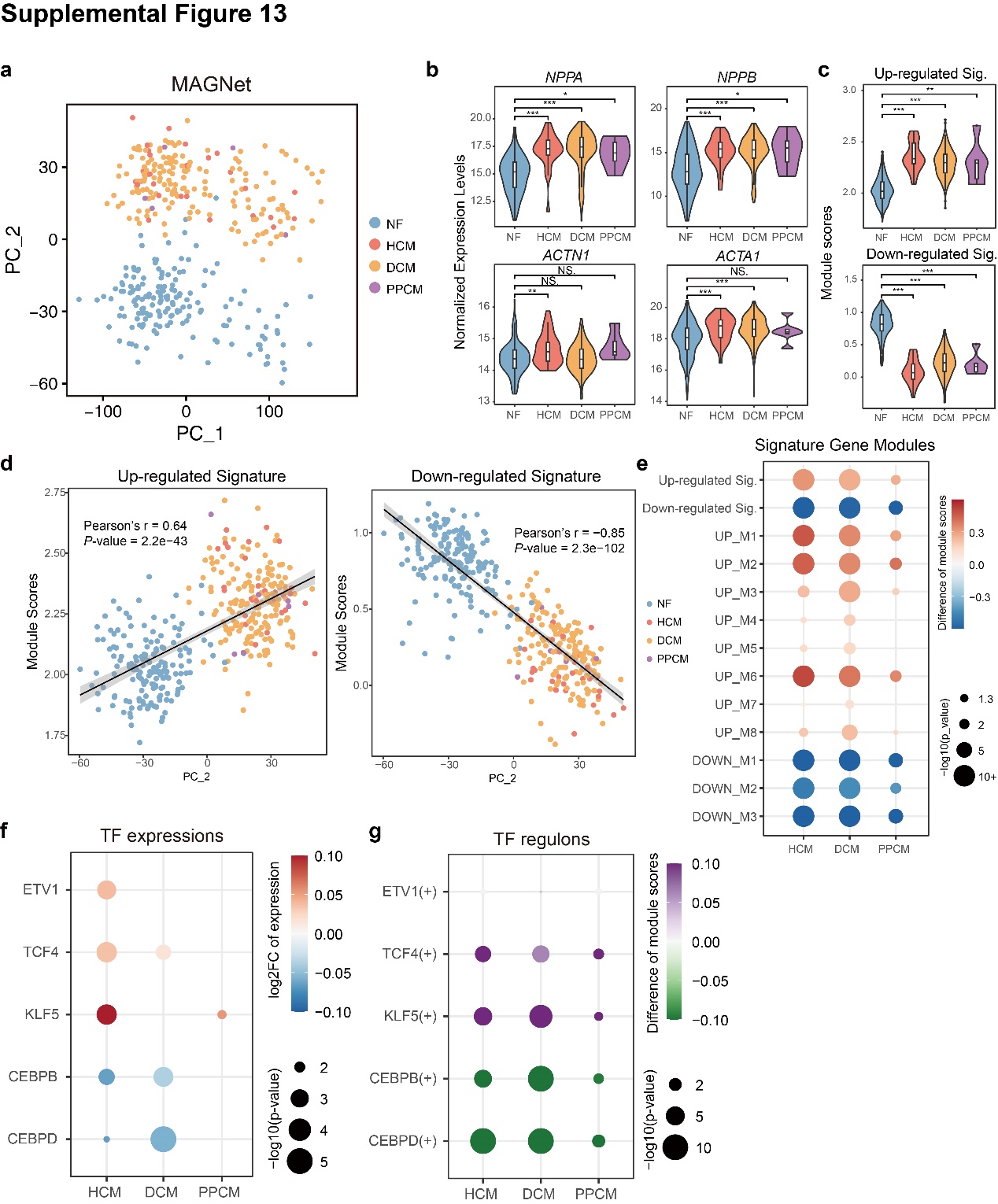
**

**Supplemental Figure 13. Analysis of bulk RNA-seq data from MAGNet.**

**a.** PCA visualizations of all MAGNet bulk RNA samples. **b.** Normalized expression levels of *NPPA*, *NPPB*, *ACTN1,* and *ACTA1* in each group of the MAGNet dataset. **c.** Module score of signature up- and down-regulated genes in MAGNet dataset. **d.** Correlation between signature up- and down-regulated gene module score and PC2 values of each sample. **e.** Relative gene module scores of signature gene modules in MAGNet dataset. Each dot was colored by the relative module score between HCM and NC, the size of the dot represented the -log_10_(*P*-value), two-sided t-test was used to determine the significance levels. **f.** Expression of *TCF4*, *ETV1*, *KLF5*, *CEBPB,* and *CEBPD* in the MAGNet dataset, colored by average expression levels. The size of the dot represented the percentage of expressed cells. **g.** Relative gene module scores of target gene regulons of *TCF4*, *ETV1*, *KLF5*, *CEBPB,* and *CEBPD* in the MAGNet dataset. Each dot was colored by the relative module score compared with NC group, the size of the dot represented the -log_10_(*P*-value), two-sided t-test was used to determine the significance levels.

**Supplemental Table 1. Information of patients.**

**Supplemental Table 2. Differentially expressed genes between cardiomyocytes of HCM and NC.**

**Supplemental Table 3. Ligand and receptor pairs were identified in cardiomyocytes of HCM and NC.**

**Supplemental Table 4. Gene Ontology enrichment results of DEGs between HCM and NC.**

**Supplemental Table 5. Signature** **up- and down-regulated genes revealed by comparative analysis.**

**Supplemental Table 6. Gene modules of signature** **up- and down-regulated genes.**

**Supplemental Table 7. Differentially expressed genes between subpopulations of HCM cardiomyocytes.**

**Supplemental Table 8. Antibody information for immunostaining.**
